# Clinical Features of COVID-19 Patients in the First Year of Pandemic: A Systematic Review and Meta-Analysis

**DOI:** 10.1177/10998004211055866

**Published:** 2021-12-04

**Authors:** Mohammed Al Maqbali, Khalid Al badi, Mohammed Al Sinani, Norah Madkhali, Geoffrey L. Dickens

**Affiliations:** 1 Department of Nursing Midwifery and Health, 373117 Northumbria University, Newcastle-Upon-Tyne, UK; 2 Al Buraimi University College, Al Buraimi, Oman; 3 105958 Al Khawarizmi International College, Al Ain, UAE; 4 Ministry of Health, Oman; 5 Reproductive and Developmental Biology, Department of Surgery and Cancer, Faculty of Medicine, 4957 Imperial College London, London, UK; 6 123285 Jazan University, Jazan, Saudi Arabia; 7 Mental Health Nursing Department of Nursing, Midwifery and Health Faculty of Health and Life Sciences, 373117 Northumbria University, Newcastle-Upon-Tyne, UK; 8 Western Sydney University, Sydney, NSW, Australia

**Keywords:** COVID-19, clinical symptoms, systematic review, meta-analysis

## Abstract

**Background:**

The new coronavirus disease (COVID-19) carries a high risk of infection and has spread rapidly around the world. However, there are limited data about the clinical symptoms globally. The purpose of this systematic review and meta-analysis is to identify the prevalence of the clinical symptoms of patient with COVID-19.

**Methods:**

A systematic review and meta-analysis were carried out. The following databases were searched: PubMed, CINAHL, MEDLINE, EMBASE, PsycINFO, medRxiv, and Google Scholar, from December 1st, 2019 to January 1st, 2021. Prevalence rates were pooled with meta-analysis using a random-effects model. Heterogeneity was tested using I-squared (I^2^) statistics.

**Results:**

A total of 215 studies, involving 132,647 COVID-19 patients, met the inclusion criteria. The pooled prevalence of the four most common symptoms were fever 76.2% (*n* = 214; 95% CI 73.9–78.5); coughing 60.4% (*n* = 215; 95% CI 58.6–62.1); fatigue 33.6% (*n* = 175; 95% CI 31.2–36.1); and dyspnea 26.2% (*n* = 195; 95% CI 24.1–28.5). Other symptoms from highest to lowest in terms of prevalence include expectorant (22.2%), anorexia (21.6%), myalgias (17.5%), chills (15%), sore throat (14.1%), headache (11.7%), nausea or vomiting (8.7%), rhinorrhea (8.2%), and hemoptysis (3.3%). In subgroup analyses by continent, it was found that four symptoms have a slight prevalence variation—fever, coughing, fatigue, and diarrhea.

**Conclusion:**

This meta-analysis found the most prevalent symptoms of COVID-19 patients were fever, coughing, fatigue, and dyspnea. This knowledge might be beneficial for the effective treatment and control of the COVID-19 outbreak. Additional studies are required to distinguish between symptoms during and after, in patients with COVID-19.

At the end of December 2019, a new coronavirus disease (COVID-19) emerged in Wuhan City, Hubei province, China, and subsequently spread worldwide (Li et al., 2020a). COVID-19 is a serious threat to human health. On January 30th, 2020, the World Health Organization (WHO) declared a public health emergency and named COVID-19 a pandemic (World Health Organization, 2020). Almost all countries and regions around the world have reported confirmed cases of COVID-19. Globally, the WHO reported more than 165 million confirmed cases worldwide, with nearly three million deaths as of May 22nd, 2021 (WHO, 2021). Identifying the main clinical symptoms is essential for early detection and for the isolation of infected patients.

A large number of studies have been published aiming to identify the clinical features of COVID-19. Several systematic reviews and meta-analyses have been published which have assessed the prevalence of baseline clinical characteristic and associated factors (Fu et al., 2020; Ghayda et al., 2020; Grant et al., 2020; Li et al., 2021; Rodriguez-Morales et al., 2020; Sun et al., 2020; Wan et al., 2020; Yang et al., 2020; Zhu et al., 2020). While such reviews are helpful, they have included relatively few studies. Moreover, a limitation was observed in those reviews in terms of geographical location and the sample characteristics included. Even though there have been a large number of publications, there is a need for an in-depth understanding of the clinical symptoms of COVID-19 which can help in tackling this pandemic and preventing future outbreaks of infectious diseases. Therefore, the aim of this study was to conduct a systematic review and meta-analysis to assess the prevalence of the symptoms associated with COVID-19.

## Methods

This systematic review and meta-analysis were undertaken according to the PRISMA standards.

### Search Strategy

A systematic literature search for the period between December 1st, 2019 and January 1st, 2021, was conducted using the following databases: PubMed, CINAHL, MEDLINE, EMBASE, PsycINFO, medRxiv, and Google Scholar. Search terms used both free text words and medical subject headings, that is, MeSH terms, to search for papers to be included in the review; that is, (MH ‘COVID-19’) OR (MH ‘coronavirus disease 2019’) OR (MH ‘severe acute respiratory syndrome coronavirus 2’) OR (MH ‘SARS-CoV-2’) OR (MH ‘2019 novel coronavirus’) OR (MH ‘2019-nCoV’) OR (MH ‘coronavirus’) OR (MH ‘corona virus’) OR ‘Wuhan pneumonia’ OR ‘COVID’ OR ‘Betacoronavirus’ OR ‘Alphacoronavirus’ OR ‘coronavir*’ AND (MH ‘clinical characteristics’) OR (MH ‘symptomatology’) OR (MH ‘Features’) OR (MH ‘Symptom*’) OR ‘signs’. In addition, the reference lists of the retrieved studies and review articles were screened to identify any further studies.

### Study Selection

Two investigators (MM; SM) performed the search, scrutinizing all titles and abstracts for eligibility against the inclusion and exclusion criteria. Any disagreements were resolved by discussion with a third investigator (KB). Studies were included in the review based on the following inclusion criteria: (1) diagnosed with COVID-19; (2) reported prevalence of clinical symptoms; (3) subjects aged 15 or older; (4) all types of settings; (5) cross-sectional or cohort surveys (only the baseline data were extracted); and (6) sample size greater than 40 to avoid selection bias from small studies. The exclusion criteria were the following: (1) protocol papers, and conference abstracts; and (2) case reports and studies with a sample size of less than 40. For any additional information, the study authors were contacted.

### Quality Assessment

Upon retrieval of the applicable studies, quality assessment was completed using the Newcastle-Ottawa Scale (NOS; Well et al., 2020). This scale consists of eight items that evaluate non-randomized studies in terms of three criteria: the selection of the participants, the comparability of study groups, and outcome assessment. The NOS uses a score system with the lowest possible score of 0 and the highest possible score of 9. The total points awarded indicate the overall quality of the study. A study was determined to be of low risk of bias when the score was 7–9, of moderate risk of bias if the score was 5–6, and of high risk of bias if the score was 0–4 (Li & Katikireddi, 2019).

### Data Analyses

The mean point of prevalence, the odds ratio (OR) with a 95% Confidence Interval (CI), was calculated as the effect size by using a random-effects model. Heterogeneity was tested using I-squared (I^2^) statistics. A value of I^2^ was considered to be low in terms of heterogeneity with 0–25%, moderate with 25–50%, and high with 50–75% (Higgins et al., 2003). The variation in terms of the prevalence of continent, NOS, and month of conducting the study was assessed by subgroup analyses if there were more than four studies in a subgroup. Meta-regression analyses were performed for moderating continuous variables (mean age, male gender, and comorbidities). A sensitivity analysis was performed by removing one study at a time to evaluate the impact of the pooled prevalence of the remaining studies (Patsopoulos et al., 2008).

Publication bias was estimated using Egger’s linear regression test (Egger et al., 1997). A *p* value of less than 0.05 was considered as indicating statistical significance. Meta-analysis was conducted using the Comprehensive Meta-Analysis software, version 2.2 (Englewood, New Jersey, USA). Forest plots were constructed using a Microsoft Excel spreadsheet constructed by Neyeloff et al. (Neyeloff et al., 2012).

## Results

**Figure 1.**
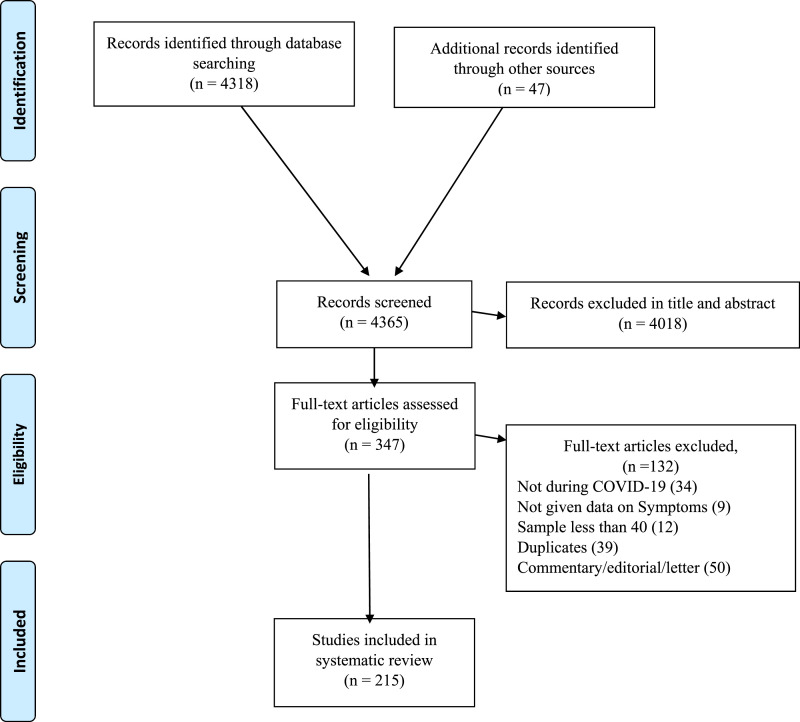
PRISMA diagram.

The database search identified 4365 papers. Of these, 4018 papers were excluded during the title and abstract screening process. A further 132 papers were excluded during full text review for the following reasons: 34 papers were not conducted during the COVID-19 period; 9 did not give data about symptoms; 39 were duplicated papers; 50 were commentary, editorial or letter papers. As such, 215 studies were identified as being eligible for meta-analysis (Figure 1 shows the PRISMA flow chart).

### General Characteristics

Two hundred and fifteen studies, involving 132,647 COVID-19 patients, were included in this meta-analysis. All studies were conducted between January 2020 and June 2020: 7 in January, 112 in February, 50 in March, 28 in April, 15 in May, and 3 in June. Seventeen preprint studies (Chen et al., 2020a, 2020b; Duan et al., 2020; Fu et al., 2020; Kuang et al., 2020; Li et al., 2020; Liu et al., 2020; Nie et al., 2020; Qi et al., 2020a, 2020b; Qin et al., 2020; Shi et al., 2020; Song et al., 2020; Tao et al., 2020; Wen et al., 2020; Xu et al., 2020; Zhao et al., 2020) were included in the analysis. All the studies included in this meta-analysis were of retrospective design. The vast majority (*n* = 208 studies) used polymerase chain reaction (PCR) to diagnose COVID-19 and two studies (Ke et al., 2020; Xie et al., 2020) used a combination of PCR and antibody tests. Five studies (Chen et al., 2020; Han et al., 2020; Xiao et al., 2020; Zheng et al., 2020; Zhou et al., 2020) did not give information about diagnostic criteria.

One hundred and fifty-one studies originated from China, 16 from the USA, 6 from Spain, 5 from Iran, 4 each from Italy, Korea, and the UK, 3 each from Belgium, France, the Kingdom of Saudi Arabia and Kuwait, 2 from Jordan, and 1 from each of the following: Bangladesh, Bulgaria, Canada, Germany, Japan, the Netherlands, Pakistan, Poland, Singapore, Somalia, and Turkey (see Supplementary Table 1 for the general characteristics of the studies).

### Quality Assessment

The studies were assessed using the NOS checklist. Thirty-nine studies were classified as having a low risk of bias, and 166 as moderate risk. The detailed results of the quality assessment of the studies included in this meta-analysis are listed in Supplementary Table 2.

### Demographic and Comorbidity Characteristics

The mean age of patients among 191 studies was 54.26 years (95% CI 52.64–55.87). All meta-analyses of prevalence estimate that in terms of the gender distribution of COVID-19, male patients accounted for 53.1% (71,194/132,546 participants, 95% CI 51.9–54.2). One hundred and ninety-three studies provided information about comorbidities. The most frequent comorbidities among patient were hypertension 26.8% (32,925/93,909 participants, 95% CI 24.7–29.1), followed by diabetes with 13.4% (18,372/124,199, 95% CI 12.4–14.4).

### Fever

**Figure 2.**
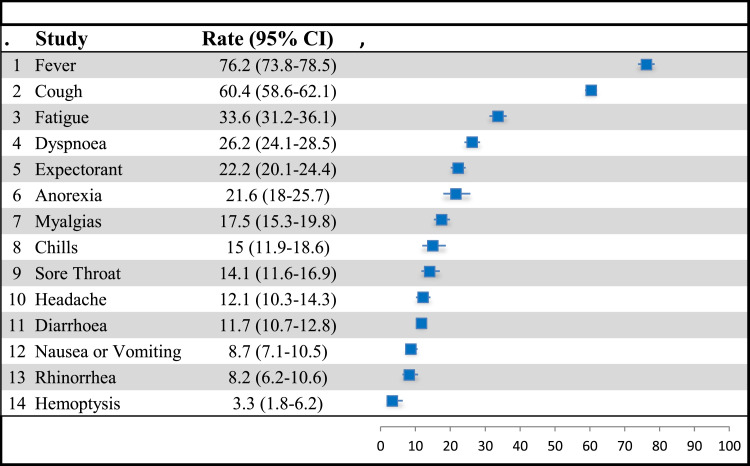
Forest plot of the prevalence of symptoms among COVID-19 patients.

Fever was estimated to occur in 214 studies. The overall pooled point estimates of prevalence for fever varied between 7.5% and 99.1%. All meta-analyses of prevalence estimates of fever reported by the 214 studies yielded a summary prevalence of 76.2% (84,823/132,436 participants, 95% CI 73.9–78.5) (Figure 2) (see Table 1). In the subgroup analyses by continent in terms of where the study was conducted, the pooled prevalence of fever was 71.7% in Asia, 68.2% in Europe, and 51.5% in North America. The pooled prevalence of fever was highest in February (82.5%) followed by January with 81.2% in (see Table 2).

**Table 1.** Prevalence of Symptoms.

	Studies No	Event	Total	Prevalence, %	95% CI	I^2^, %	Sensitivity, %	Egger Test
Age	191			54.26	52.6–55.8			
Male	214	71,194	132,546	53.1	51.9–54.2	93	1	0.11
Comorbidities								
Hypertension	171	32,925	93,909	26.8	24.7–29.1	98.2	1	0.000
Diabetes	182	18,372	124,199	13.4	12.4–14.4	95.4	0.5	0.02
Cardiovascular	173	19,086	123,321	9.8	8.5–11.1	98.4	0.5	0.000
Respiratory	163	9475	114,861	6	5.3–6.9	96.9	0.5	0.000
Renal	161	5434	111,313	3.6	3–4.4	97.4	0.5	0.009
Liver	110	8966	103,151	3.2	2.2–4.5	99.1	0.5	0.000
Malignancy	125	4863	100,506	3.4	2.8–4	96.5	0.5	0.000
Cerebrovascular	77	3685	38,468	5.4	4.1–7	97.9	0.5	0.000
Fever	214	84,823	132,436	76.2	73.8–78.5	98.9	1	0.000
Cough	215	73,778	132,647	60.4	58.6–62.1	97.2	0.5	0.000
Fatigue	175	28,306	78,973	33.6	31.2–36.1	97.7	1	0.01
Dyspnea	195	46,681	127,715	26.2	24.1–28.5	98.6	0.5	0.000
Expectorant	102	14,159	65,275	22.2	20.1–24.4	97	0.5	0.76
Anorexia	63	4126	19,004	21.6	18–25.7	97	1	0.17
Myalgias	130	16,762	91,491	17.5	15.3–19.8	98.3	0.5	0.29
Chills	45	2728	17,303	15	11.9–18.6	97.1	1	0.3
Sore throat	80	5389	41,810	14.1	11.6–16.9	97.8	1	0.02
Headache	141	9164	63,999	12.1	10.3–14.3	98	1	0.00
Diarrhea	186	14,008	95,345	11.7	10.7–12.8	96.1	0.5	0.000
Nausea or vomiting	95	4093	41,319	8.7	7.1–10.5	97	0.5	0.000
Rhinorrhea	76	3452	30,150	8.2	6.2–10.6	97.9	1	0.00
Hemoptysis	41	12,578	49,942	3.3	1.8–6.2	99	0.5	0.000

**Table 2.** Subgroups Analyses and Meta-Regression.

Subgroups Analysis	Studies No	Prevalence	CI 95%	*I* ^*2*^	*p*	Meta-Regression	*B*	z	*p*
Fever									
Continent									
Asia	170	71.7	59.1–81.6	98.3	<0.001	Male	0.001	1.77	0.54
North America	17	51.5	40.2–62.7	98.8	<0.001	Mean age	0.042	0.61	0.07
Europe	26	68.2	58.1–76.9	99.8	<0.001				
						Hypertension	0.002	0.46	0.64
NOS						Diabetes	0.000	0.03	0.97
Moderate	175	76.7	74.5–78.8	95.6	<0.001	Cardiovascular	−0.011	−1.14	0.25
Low	39	72.5	66.1–78	99.7	<0.001	Respiratory	0.004	0.79	0.43
						Renal	−0.008	−1.57	0.11
Month						Liver	−0.001	−0.36	0.72
January	7	81.6	71.6–88.6	95.4	<0.001	Malignancy	−0.004	−0.75	0.45
February	112	82.5	79.5–85	97.5	<0.001	Cerebrovascular	0.003	0.48	0.63
March	49	71.8	67.4–75.9	98	<0.001				
April	28	64.9	55.9–73	99	<0.001				
May	15	51.1	41.6–60.6	97.9	<0.001				
Cough									
Subgroups analysis						Meta-regression			
Continent									
Asia	171	54.7	54.3–55	95	<0.001	Male	−0.001	−0.77	0.44
North America	26	57.7	57.2–58.2	98	<0.001	Mean age	0.000	−0.01	1.00
Europe	17	49	47.9–50.1	99	<0.001				
						Hypertension	0.002	0.79	0.43
NOS						Diabetes	−0.004	−1.14	0.25
Moderate	39	55.8	52.2–59.4	95	<0.001	Cardiovascular	−0.007	−1.12	0.26
Low	176	61.6	59.2–63.9	99	<0.001	Respiratory	0.006	1.91	0.06
Month						Renal	−0.001	−0.35	0.73
January	7	63.7	56.7–70.1	89.5	<0.001	Liver	0.000	0.32	0.75
February	112	61.2	58.4–63.8	95	<0.001	Malignancy	0.001	0.34	0.73
March	50	57.8	54.4–61.2	96.8	<0.001	Cerebrovascular	0.001	0.21	0.83
April	28	63.6	57.9–69	98	<0.001				
May	15	55	42.4–66.9	98.8	<0.001				
Fatigue									
Subgroups analysis						Meta-regression			
Continent									
Asia	142	31.7	29.1–34.4	96.7	<0.001	Male	−0.002	−1.16	0.25
North America	11	36.7	29–45.1	96	<0.001	Mean age	0.040	1.73	0.08
Europe	21	45.7	39–52.6	98.7	<0.001				
						Hypertension	0.003	0.84	0.40
NOS						Diabetes	−0.007	−1.68	0.09
Moderate	145	34.5	31.4–37.7	99	<0.001	Cardiovascular	−0.001	−0.18	0.86
Low	30	29.4	24.7–34.5	96	<0.001	Respiratory	0.005	0.91	0.36
						Renal	−0.002	−0.57	0.57
Month						Liver	−0.002	−0.90	0.37
January	7	30.2	24.2–36.9	89.5	<0.001	Malignancy	−0.002	−0.44	0.66
February	98	34.1	31.1–37.2	95.8	<0.001	Cerebrovascular	0.001	0.24	0.81
March	39	29	23.3–35.5	98.6	<0.001				
April	18	30.5	22.3–40	98.5	<0.001				
May	11	47.8	34–61.9	98	<0.001				
Dyspnea									
Subgroups analysis						Meta-regression			
Continent									
Asia	154	22.5	20.3–25	97.9	<0.001	Male	−0.001	−0.80	0.42
North America	14	41.6	26.8–57.9	99	<0.001	Mean age	0.076	3.49	0.00
Europe	26	42.4	36.6–48.5	99	<0.001				
						Hypertension	−0.003	−0.70	0.49
NOS						Diabetes	−0.009	−1.78	0.08
Moderate	157	26.2	23.4–29.2	96.8	<0.001	Cardiovascular	−0.001	−0.09	0.93
Low	38	25.5	21.4–30.1	99.5	<0.001	Respiratory	0.015	3.13	0.00
						Renal	0.001	0.29	0.78
Month						Liver	0.003	1.75	0.08
January	6	19.2	15.8–23.1	74.5	0.001	Malignancy	0.004	0.76	0.45
February	103	23.5	20.2–27.2	97.5	<0.001	Cerebrovascular	−0.002	−0.45	0.65
March	43	26.1	22.2–30.5	98.2	<0.001				
April	26	33.2	27–40.1	98.8	<0.001				
May	14	34.6	23.4–48.4	98.7	<0.001				
Expectorant									
Subgroups analysis						Meta-regression			
Continent									
Asia	90	29.8	29.3–30.4	95.4	<0.001	Male	0.000	−0.12	0.90
North America	2	—	—	—	—	Mean age	−0.004	−0.12	0.91
Europe	10	17.5	17–17.9	96.4	<0.001				
						Hypertension	−0.006	−0.53	0.60
NOS						Diabetes	0.013	0.80	0.42
Moderate	78	22.4	19.8–25.6	93	<0.001	Cardiovascular	−0.006	−0.57	0.57
Low	24	21.3	17.6–25.6	99	<0.001	Respiratory	−0.046	−1.57	0.12
						Renal	0.037	1.31	0.19
Month						Liver	−0.017	−0.45	0.65
January	5	28.4	21.2–36.9	92.1	<0.001	Malignancy	−0.013	−1.19	0.24
February	58	24.5	21.9–27.3	92.9	<0.001	Cerebrovascular	0.074	1.67	0.10
March	25	20.6	16.7–25.1	98	<0.001				
April	8	20.3	11.2–33.8	98.5	<0.001				
May	5	10.3	4.2–23.2	98	<0.001				
Anorexia									
Subgroups analysis						Meta-regression			
Continent									
Asia	54	20	16–24.6	96.6	<0.001	Male	0.029	3.17	0.00
Europe	6	39.5	25.7–55.2	98	<0.001	Mean age	0.144	1.56	0.12
NOS						Hypertension	−0.080	−1.94	0.05
Moderate	53	16.3	10.3–25	98.6	<0.001	Diabetes	0.029	0.71	0.48
Low	10	22.6	18.3–27.7	96	<0.001	Cardiovascular	−0.018	−0.57	0.57
						Respiratory	0.032	0.52	0.61
						Renal	0.063	0.85	0.40
Month						Liver	−0.334	−2.23	0.03
February	39	20.6	16.4–25.7	96	<0.001	Malignancy	−0.026	−0.12	0.90
March	17	23.4	14.9–34.8	97.6	<0.001	Cerebrovascular	0.053	0.61	0.55
April	5	31	19.2–46.1	98	<0.001				
Myalgias									
Subgroups analysis						Meta-regression			
Continent									
Asia	101	15.4	13.8–17.3	96	<0.001	Male	−0.003	−1.00	0.32
North America	13	26.6	17–39.1	98.8	<0.001	Mean age	−0.048	−1.05	0.30
Europe	16	26.7	14.7–13.6	99.5	<0.001				
						Hypertension	0.000	−0.02	0.99
NOS						Diabetes	−0.011	−1.26	0.21
Moderate	103	18.2	15.6–21.2	96.8	<0.001	Cardiovascular	0.012	1.05	0.30
Low	27	14.7	11–19.2	99	<0.001	Respiratory	0.003	0.16	0.87
						Renal	0.006	0.22	0.83
Month						Liver	0.026	1.05	0.29
February	66	14.9	12.8–17.3	94	<0.001	Malignancy	0.019	0.30	0.77
March	31	16.3	12.5–21	97.7	<0.001	Cerebrovascular	−0.011	−0.59	0.55
April	18	30.6	21.9–40.9	99	<0.001				
May	10	17.3	7.5–35	99	<0.001				
Chills									
Subgroups analysis						Meta-regression			
Continent									
Asia	38	13	10.4–16.3	96.4	<0.001	Male	0.001	0.19	0.85
North America	—	—	—	—	<0.001	Mean age	−0.020	−0.23	0.82
Europe	—	—	—	—	<0.001				
						Hypertension	−0.011	−1.13	0.26
NOS						Diabetes	0.005	0.55	0.58
Moderate	37	15.3	11.4–20.1	96.5	<0.001	Cardiovascular	—	—	—
Low	8	13.1	9–18.5	97.7	<0.001	Respiratory	—	—	—
						Renal	—	—	—
Month						Liver	—	—	—
February	18	13.3	10.7–16.3	83.6	<0.001	Malignancy	—	—	—
March	11	15.9	9.8–24.7	96.9	<0.001	Cerebrovascular	—	—	—
April	8	16.2	9–27.4	98.2	<0.001				
May	5	24.4	12.8–41.4	95.3	<0.001				
Sore throat									
Subgroups analysis						Meta-regression			
Continent									
Asia	60	12.3	10.7–14.4	93	<0.001	Male	−0.001	−0.18	0.85
North America	9	21.6	14–31.8	96.6	<0.001	Mean age	−0.090	−1.09	0.28
Europe	10	24.1	10.2–47	99.5	<0.001				
						Hypertension	−0.002	−0.08	0.94
NOS						Diabetes	0.008	0.24	0.81
Moderate	66	15.5	13.3–18.1	94.6	<0.001	Cardiovascular	0.009	0.35	0.73
Low	14	10.3	5.5–18.5	99	<0.001	Respiratory	−0.011	−0.09	0.93
						Renal	−0.012	−0.07	0.94
Month						Liver	−0.001	−0.01	0.99
February	37	11.2	9–13.8	93.9	<0.001	Malignancy	−0.005	−0.18	0.86
March	16	12.4	7–21	98.5	<0.001	Cerebrovascular	0.061	0.51	0.61
April	16	25.4	18.8–33.3	97.7	<0.001				
May	5	19.3	12.3–29.1	93.2	<0.001				
Headache									
Subgroups analysis						Meta-regression			
Continent									
Asia	115	10.2	9.2–28.3	94	<0.001	Male	0.001	0.28	0.78
North America	10	23.6	14.3–36.4	98	<0.001	Mean age	−0.063	−1.83	0.07
Europe	15	27.1	14.1–45.8	99.6	<0.001				
						Hypertension	−0.009	−1.15	0.25
NOS						Diabetes	−0.002	−0.24	0.81
Moderate	112	12.9	10.7–15.5	96.9	<0.001	Cardiovascular	0.012	0.96	0.34
Low	29	9.6	6.7–13.6	99	<0.001	Respiratory	0.004	0.54	0.59
						Renal	0.008	1.11	0.27
Month						Liver	−0.018	−0.57	0.57
January	6	13.3	10.1–17.4	81.3	<0.001	Malignancy	0.005	0.70	0.48
February	73	9.4	8.3–10.7	88.3	<0.001	Cerebrovascular	0.002	0.19	0.85
March	33	11	8.4–14.4	97.7	<0.001				
April	18	25.6	17.8–35.4	98.7	<0.001				
May	10	20.3	9.1–39.1	98	<0.001				
Diarrhea									
Subgroups analysis						Meta-regression			
Continent									
Asia	147	16.7	9.2–28.3	93	<0.001	Male	−0.002	−2.23	0.03
North America	16	18.3	12.2–26.6	98	<0.001	Mean age	−0.014	−0.6	0.55
Europe	22	16.8	13.6–20.6	98	<0.001				
						Hypertension	0.003	0.64	0.52
NOS						Diabetes	0.002	0.45	0.66
Moderate	150	11.7	10.3–13.2	94	<0.001	Cardiovascular	−0.003	−0.38	0.71
Low	36	10.9	9.1–13.1	98.5	<0.001	Respiratory	0.002	0.42	0.68
						Renal	−0.002	−0.32	0.75
Month						Liver	−0.003	−0.66	0.51
January	7	5.2	3.7–7.5	67	0.006	Malignancy	−0.003	−0.05	0.96
February	94	10.7	9.3–12.2	93.2	<0.001	Cerebrovascular	0.001	0.19	0.85
March	43	11.2	9.7–12.9	92.9	<0.001				
April	26	17.4	14.1–21.2	95.9	<0.001				
May	13	15.3	8.7–25.4	98	<0.001				
Nausea or vomiting									
Subgroups analysis						Meta-regression			
Continent									
Asia	74	7.1	5.7–8.8	94.8	<0.001	Male	−0.003	−1.82	0.07
North America	8	20.1	14.7–26.8	93.3	<0.001	Mean age	−0.040	−1.59	0.11
Europe	12	13.3	8.1–21.2	98.6	<0.001				
						Hypertension	0.000	−0.09	0.93
NOS						Diabetes	−0.010	−1.86	0.06
Moderate	75	7	4.8–10.2	98.5	<0.001	Cardiovascular	0.030	2.56	0.01
Low	20	9	7.1–11.4	95.8	<0.001	Respiratory	−0.003	−0.50	0.61
						Renal	0.014	2.57	0.01
Month						Liver	−0.001	−0.26	0.80
February	50	7.5	5.9–9.6	93.4	<0.001	Malignancy	0.001	0.15	0.88
March	21	9.4	6–14.4	97.8	<0.001	Cerebrovascular	−0.017	−2.49	0.01
April	14	13.4	9.6–18.4	96.4	<0.001				
May	5	10.2	3.4–27.1	98	<0.001				
Rhinorrhea									
Subgroups analysis						Meta-regression			
Continent									
Asia	61	5.9	4.6–7.5	94.9	<0.001	Male	−0.002	−0.36	0.72
North America	7	28.4	20.5–38	94.6	<0.001	Mean age	−0.133	−1.25	0.21
Europe	8	25.1	12.1–44.8	99	<0.001				
						Hypertension	−0.017	−0.77	0.44
NOS						Diabetes	−0.006	−0.40	0.69
Moderate	59	9.2	7.2–11.7	95.5	<0.001	Cardiovascular	0.034	1.75	0.08
Low	17	5.9	2.9–11.7	99	<0.001	Respiratory	−0.045	−0.86	0.39
						Renal	0.046	0.70	0.49
Month						Liver	0.033	0.64	0.53
February	41	5.5	4.2–7.1	91	<0.001	Malignancy	0.167	1.35	0.18
March	17	11.1	7.3–16.6	96	<0.001	Cerebrovascular	0.015	0.31	0.76
April	10	21.6	11.9–35.9	98.9	<0.001				
Hemoptysis									
Subgroups analysis						Meta-regression			
Continent									
Asia	3.6	3.2	1.6–3.6	98.7	<0.001	Male	−0.006	−1.58	0.11
						Mean age	−0.110	−0.84	0.40
NOS									
Moderate	30	4.1	2.6–6.3	92.5	<0.001	Hypertension	0.001	0.08	0.94
Low	11	2.1	0.5–8	99.6	<0.001	Diabetes	−0.020	−0.99	0.32
						Cardiovascular	0.010	1.17	0.24
Month						Respiratory	0.020	0.69	0.49
February	21	2.8	2–4	78.4	<0.001	Renal	0.016	1.53	0.13
March	8	6.9	2.7–16.7	97.7	<0.001	Liver	−0.041	−1.74	0.08
April	6	4	0.8–17.2	99.2	<0.001	Malignancy	−0.135	−1.24	0.22
						Cerebrovascular	0.067	1.26	0.21

### Coughing

Coughing was identified in 215 studies. The overall pooled point estimates of the prevalence of coughing varied between 16% and 93.3%. All meta-analyses of prevalence estimates of coughing reported by the 215 studies yielded a summary prevalence of 60.4% (73,778/132,647 participants, 95% CI 58.6–62.1). A subgroups analysis by continent showed a coughing prevalence of 57.7% for North America, 54.7% for Asia, and 49% for Europe.

### Fatigue

Fatigue was estimated in 175 studies. The overall pooled point estimates of prevalence for fatigue varied between 3.4% and 90.6%. All meta-analyses of prevalence estimates of fatigue reported by the 175 studies yielded a summary prevalence of 33.6% (28,306/78,973 participants, 95% CI 31.2–36.1). The pooled prevalence rates with regard to fatigue were 45.7% for Europe, 36.7% for North America, and 31.7% for Asia.

### Dyspnea

Dyspnea was estimated in 195 studies. The overall pooled point estimates of prevalence for dyspnea varied between 1% and 99%. All meta-analyses of the prevalence estimates of dyspnea reported by the 195 studies yielded a summary prevalence of 26.2% (46,681/127,715 participants, 95% CI 24.1–28.5). In the subgroup analyses by continent, the pooled prevalence of dyspnea was 42.4% for Europe, 41.6% for North America, and 22.5% for Asia. The pooled prevalence of dyspnea was highest in May (34.6%). In meta-regression analyses, the mean age and patients with respiratory disease were significantly associated with the dyspnea prevalence rate (*p* < 0.001).

### Expectorant

Expectorant was estimated in 102 studies. The overall pooled point estimates of prevalence for expectorant varied between 2.2% and 56.5%. All meta-analyses of prevalence estimates of expectorant reported by the 102 studies yielded a summary prevalence of 22.2% (14,159/65,275 participants, 95% CI 20.1–24.4). A subgroups analysis by continent showed the expectorant prevalence of 29.8% for Asia and 17.5% for Europe.

### Anorexia

Anorexia was estimated in 63 studies. The overall pooled point estimates of prevalence for anorexia varied between 3% and 86%. All meta-analyses of prevalence estimates of anorexia reported by the 63 studies yielded a summary prevalence of 21.6% (4126/19,004 participants, 95% CI 18–25.8). In the subgroup analyses, the prevalence of anorexia was reported in 39.5% of the studies conducted in Europe compared to 20% of the studies conducted in Asia. The male gender, hypertension, and liver disease patients were significantly associated with the anorexia symptoms prevalence rate (*p* < 0.05).

### Myalgias

Myalgias were estimated in 130 studies. The overall pooled point estimates of prevalence for myalgias varied between 0.8% and 65.3%. All meta-analyses of prevalence estimates of myalgias reported by the 130 studies yielded a summary prevalence of 17.5% (16,762/91,491 participants, 95% CI 15.3–19.8). In the subgroup analyses, the prevalence of myalgias was similarly reported by studies from Europe (36.7%) and North America (26.6%), whereas it was lower in Asia (15.4%). The pooled prevalence of myalgia was highest in April (30.6%).

### Chills

Chills were estimated in 45 studies. The overall pooled point estimates of prevalence for chills varied between 1.6% and 53.3%. All meta-analyses of the prevalence estimates of chills reported by the 45 studies yielded a summary prevalence of 15% (2728/17,303 participants, 95% CI 11.9–18.6).

### Sore Throat

Sore throat was estimated in 80 studies. The overall pooled point estimates of prevalence for sore throat varied between 1.2% and 57.4%. All meta-analyses of prevalence estimates of sore throat reported by the 80 studies yielded a summary prevalence of 14.1% (5389/41,810 participants, 95% CI 11.6–16.9). In the subgroup analyses, the highest prevalence of sore throat was in Europe (24.1%) and North America (21.6%) compared to studies conducted in Asia (12.3%). Pooled prevalence of sore throat was highest in April (25.4%).

### Headache

Headache was estimated in 141 studies. The overall pooled point estimates of prevalence for headache varied between 1.5% and 75%. All meta-analyses of prevalence estimates of headache reported by the 141 studies yielded a summary prevalence of 12.1% (9164/63,999 participants, 95% CI 10.3–14.3). A subgroup analysis by continent showed the headache prevalence of 27.1% for Europe, 23.7% for North America, and 10.2% for Asia. The month of April showed the highest prevalence of headache (25.6%).

### Diarrhea

Diarrhea was estimated in 186 studies. The overall pooled point estimates of prevalence for diarrhea varied between 1% and 50.3%. All meta-analyses of prevalence estimates of diarrhea reported by the 186 studies yielded a summary prevalence of 11.7% (14,008/95,345 participants, 95% CI 10.7–12.8). The prevalence of diarrhea in North America was 18.3%, in Europe 16.8%, and in Asia 16.7%. Male patients were significantly associated with the prevalence of diarrhea symptoms (*p* = 0.03).

### Nausea or Vomiting

Nausea or vomiting was estimated in 95 studies. The overall pooled point estimates of prevalence for nausea or vomiting varied between 1% and 96.3%. All meta-analyses of prevalence estimates of nausea or vomiting reported by the 95 studies yielded a summary prevalence of 8.7% (4093/41,319 participants, 95% CI 7.1–10.5). A subgroups analysis by continent showed that nausea or vomiting prevalence was highest North America (20.1%), compared to Europe (13.3%) and Asia (7.1%). In meta-regression analyses, patients with cardiovascular, renal, and cerebrovascular disease were significantly associated with the nausea or vomiting prevalence rate (*p* < 0.001).

### Rhinorrhea

Rhinorrhea was estimated in 76 studies. The overall pooled point estimates of prevalence for rhinorrhea varied between 0.2% and 60.1%. All meta-analyses of prevalence estimates of rhinorrhea reported by the 76 studies yielded a summary prevalence of 8.2% (3452/30,150 participants, 95% CI 6.2–10.6). In the subgroup analyses, the prevalence of rhinorrhea was higher in both North America (28.4%) and Europe (25.1%), whereas in Asia it was reported as 5.9%.

### Hemoptysis

Hemoptysis was estimated in 41 studies. The overall pooled point estimates of prevalence for hemoptysis varied between 0.1% and 41.4%. All meta-analyses of the prevalence estimates of hemoptysis reported by the 41 studies yielded a summary prevalence of 3.3% (12,578/49,942 participants, 95% CI 1.8–6.2).

### Sensitivity Analysis

Sensitivity analysis was conducted with regard to all subgroups by excluding one study each time. This demonstrated that there were no differences in the overall estimation by more or less than 1%.

### Publication Bias

Publication bias was assessed using Egger’s regression test. Evidence of bias was found with regard to the following: Coughing (*n* = 215; *p <* 0.001); Fever (*n* = 214; *p <* 0.001); Dyspnea (*n* = 195; *p <* 0.001); Diarrhea (*n* = 186; *p <* 0.001); Fatigue (*n* = 175; *p* = 0.01); Headache (*n* = 141; *p <* 0.001); Nausea or Vomiting (*n* = 95; *p <* 0.001); Sore Throat (*n* = 80; *p* = 0.01); Rhinorrhea (*n* = 76; *p <* 0.001); Hemoptysis (*n* = 41; *p <* 0.001). On the other hand, Chills (*n* = 186; *p* = 0.15); Myalgias (*n* = 130; *p* = 0.15); Expectorant (*n* = 102; *p* = 0.37); and Anorexia (*n* = 63; *p* = 0.18) did not show the presence of publication bias.

## Discussion

COVID-19 is viewed as a major threat to public health due to the incredible damage it is doing to the medical services and the economies of almost all countries across the global (Wiebers & Feigin, 2020). Given that it is a new infectious disease, assessing the clinical signs and symptoms of COVID-19 is imperative for early detection and for the isolation of infected patients in order to reduce the spread of the disease, and to identify appropriate management strategies. This meta-analysis was undertaken to estimate the aggregate prevalence of clinical signs and symptoms of COVID-19 patients.

This meta-analysis found that there was a minor variation in terms of COVID-19 patients between male (53.7%) and female (46.3%). This is consistent with other meta-analyses that have found that there were slightly more male than female patients (Li et al., 2021; Zhu et al., 2020). However, in contrast, a meta-analysis conducted by Peckham et al. (2020) involving 3,111,714 subjects found no difference with regard to the number of males and females with COVID-19. This difference might be linked to sex hormones and X-linked genes that may inactivate immunity with regard to responses (Agrawal et al., 2021). Clearly, the gender disparity with regard to COVID-19 is not fully understood.

In this meta-analysis, the most frequently reported clinical features of COVID-19 were fever, coughing, fatigue, and dyspnea (76.2%, 60.4%, 33.6%, and 26.2%. respectively). These results are lower by between approximately 5% and 20% compared to other meta-analyses (Fu et al., 2020; Ghayda et al., 2020; Grant et al., 2020; Li et al., 2021; Rodriguez-Morales et al., 2020; Sun et al., 2020; Wan et al., 2020; Yang et al., 2020; Zhu et al., 2020). For example, Yang et al. (2020) undertook a meta-analysis of seven studies which included 1576 COVID-19 patients, and found: fever (91%), coughing (67%), fatigue (51%), and dyspnea (30%). This could be explained by this meta-analysis including a relatively small sample of COVID-19 patients ranging from 656 (Rodriguez-Morales et al., 2020) to 50,466 (Sun et al., 2020). In contrast, the meta-analysis reported in this paper involved 132,647 COVID-19 patients, which might have caused these differences in symptom prevalence. Another possible reason is that three reviews (Ghayda et al., 2020; Li et al., 2021; Zhu et al., 2020) included pediatric COVID-19 patients in their analysis. Ding et al. (2020) conducted a meta-analysis with 387 children diagnosed with COVID-19 and reported the prevalence of symptoms as follows: fever 51.2%, coughing 37%, and dyspnea 4%, all of which are less frequent than is the case with adult patients. In our meta-analysis, only those above 15 years of age were included.

The results of the current meta-analyses are even lower when compared with studies which reported symptoms at onset of fever, coughing, fatigue, and dyspnea for the Middle East Respiratory Syndrome (MERS) and Severe Acute Respiratory Syndrome (SARS) epidemics (Assiri et al., 2016; Azhar et al., 2019; Cleri et al., 2010; Yin & Wunderink, 2018; Z. Zhao et al., 2003). Furthermore, in sub-group analyses by continent, differences were found in the reported prevalence of symptoms. Studies conducted in Asia reported the highest prevalence of fever, expectorant, chills, and hemoptysis. In Europe, fatigue, dyspnea, anorexia, myalgias, sore throat, and headache were ranked as the most prevalent, whereas studies conducted in North America reported the highest prevalence of coughing, nausea or vomiting, and rhinorrhea. The prevalence of diarrhea was approximately the same in three continents. This variation of the symptoms between continents may be explained by variations between the patients in terms of social conditions, environment, and geographical location.

The findings of this analysis suggested that four symptoms have slight variations in prevalence between the continents—fever, coughing, fatigue, and diarrhea. However, it is recommended that every country should have its own symptoms list to evaluate patients.

The meta-regression analysis also revealed that male patients were significantly associated with a pooled estimation of anorexia, diarrhea, and nausea or vomiting. In addition, mean age and respiratory disease yielded a higher prevalence of dyspnea. These finding warrant further examination.

The major strength of this meta-analysis is the large sample size of over 132,647 subjects drawn from 215 studies which estimated the symptom onset of patients diagnosed with COVID-19. However, there are several potential limitations to this meta-analysis. First, this review searched medRxiv’s preprint studies which, at the time of searching, were not peer reviewed. This might introduce publication bias. Second, there is a possibility that some studies have not been included in this meta-analysis, even though this analysis used different MeSH terms and several databases. In addition, only studies published, unpublished, or translated into English were included in this analysis. Third, around 151 of the studies originated from China, while the other 64 studies originated from 22 countries. Two studies from Spain included 25,615 subjects. Fourth, there were insufficient data available with regard to the demographic and clinical characteristics, so not all information could be eliminated thoroughly. Finally, all findings were derived from retrospective designs, which means that we cannot rule out selection bias.

This is a systematic review and meta-analysis reporting pooled estimates with regard to the prevalence of symptoms associated with COVID-19. Thus, the findings can be used to help healthcare professionals and policy makers identify and monitor patients as part of the early screening process of COVID-19 patients. This might help ensure the appropriate utilization of healthcare resources, which in turn might help to reduce the severity of the impact of COVID-19 and be beneficial when it comes to effective management and treatment.

## Conclusion

This meta-analysis has been undertaken to estimate the aggregate prevalence of the clinical signs and symptoms of COVID-19 patients. This meta-analysis found that the most prevalent symptoms with regard to COVID-19 patients were fever, coughing, fatigue, and dyspnea. This knowledge might be beneficial for the effective treatment and control of the COVID-19 pandemic. Additional studies are required to distinguish between symptoms during and after in patients with COVID-19.

## Supplemental Material

sj-pdf-1-brn-10.1177_10998004211055866 – Supplemental Material for Clinical Features of COVID-19 Patients in the First Year of Pandemic: A Systematic Review and Meta-Analysis Click here for additional data file. Supplemental Material, sj-pdf-1-brn-10.1177_10998004211055866 for Clinical Features of COVID-19 Patients in the First Year of Pandemic: A Systematic Review and Meta-Analysis by Mohammed Al Maqbali, Khalid Al badi, Mohammed Al Sinani, Norah Madkhali and Geoffrey L. Dickens in Biological Research For Nursing

sj-pdf-2-brn-10.1177_10998004211055866 – Supplemental Material for Clinical Features of COVID-19 Patients in the First Year of Pandemic: A Systematic Review and Meta-Analysis Click here for additional data file. Supplemental Material, sj-pdf-2-brn-10.1177_10998004211055866 for Clinical Features of COVID-19 Patients in the First Year of Pandemic: A Systematic Review and Meta-Analysis by Mohammed Al Maqbali, Khalid Al badi, Mohammed Al Sinani, Norah Madkhali and Geoffrey L. Dickens in Biological Research For Nursing

## References

[bibr1-10998004211055866] Agrawal H. Das N. Nathani S. Saha S. Saini S. Kakar S. S. Roy P. (2021). An assessment on impact of COVID-19 infection in a gender specific manner. Stem Cell Reviews and Reports, 17(1), 94-112. 10.1007/s12015-020-10048-z. 33029768 PMC7541100

[bibr2-10998004211055866] Assiri A. Abedi G. R. Saeed A. A. B. Abdalla M. A. al-Masry M. Choudhry A. J. Lu X. Erdman D. D. Tatti K. Binder A. M. Rudd J. Tokars J. Miao C. Alarbash H. Nooh R. Pallansch M. Gerber S. I. Watson J. T. (2016). Multifacility outbreak of middle east respiratory syndrome in Taif, Saudi Arabia. Emerging Infectious Diseases, 22(1), 32–40. 10.3201/eid2201.151370 26692003 PMC4696715

[bibr3-10998004211055866] Azhar E. I. Hui D. S. C. Memish Z. A. Drosten C. Zumla A. (2019). The middle east respiratory syndrome (MERS). Infectious Disease Clinics of North America, 33(4), 891–905. 10.1016/j.idc.2019.08.001 31668197 PMC7127753

[bibr4-10998004211055866] Chen M. Fan Y. Wu X. Zhang L. Guo T. Deng K. Cao J. Luo H. He T. Gong Y. Wang H. Wan J. Wang X. Lu Z. (2020a). Clinical characteristics and risk factors for fatal outcome in patients with 2019-coronavirus infected disease (COVID-19) in Wuhan, China [SSRN Scholarly Paper]. 10.2139/ssrn.3546069

[bibr5-10998004211055866] Chen Z. Hu J. Zhang Z. Jiang S. Wang T. Shi Z. Zhang Z. (2020b). Caution: The clinical characteristics of COVID-19 patients at admission are changing. MedRxiv. 10.1101/2020.03.03.20030833

[bibr6-10998004211055866] Chen F. Sun W. Sun S. Li Z. Wang Z. Yu L. (2020c). Clinical characteristics and risk factors for mortality among inpatients with COVID-19 in Wuhan, China. Clinical and Translational Medicine, 10(2):e40. 10.1002/ctm2.40 32508024 PMC7300688

[bibr7-10998004211055866] Cleri D. J. Ricketti A. J. Vernaleo J. R. (2010). Severe acute respiratory syndrome (SARS). Infectious Disease Clinics of North America, 24(1), 175–202. 10.1016/j.idc.2009.10.005 20171552 PMC7135483

[bibr8-10998004211055866] Ding Y. Yan H. Guo W. (2020). Clinical characteristics of children with COVID-19: A meta-analysis. Frontiers in Pediatrics, 8, 431. 10.3389/fped.2020.00431 32719759 PMC7350605

[bibr9-10998004211055866] Duan L. Zhang S. Guo M. Zhou E. Fan J. Wang X. Wang L. Wu F. Jin Y. (2020). Epidemiological and clinical characteristics in patients with SARS-CoV-2 antibody negative probable COVID-19 in Wuhan. MedRxiv. 10.1101/2020.06.18.20134619

[bibr10-10998004211055866] Egger M. Smith G. D. Schneider M. Minder C. (1997). Bias in meta-analysis detected by a simple, graphical test. British Medical Journal, 315(7109), 629–634. 10.1136/bmj.315.7109.629 9310563 PMC2127453

[bibr11-10998004211055866] Fu S. Fu X. Song Y. Li M. Pan P. Tang T. Zhang C. Jiang T. Tan D. Fan X. Sha X. Ma J. Huang Y. Li S. Zheng Y. Qian Z. Xiong Z. Xiao L. Long H. Ouyang Y. (2020a). Virologic and clinical characteristics for prognosis of severe COVID-19: A retrospective observational study in Wuhan, China. MedRxiv. 10.1101/2020.04.03.20051763

[bibr12-10998004211055866] Fu L. Wang B. Yuan T. Chen X. Ao Y. Fitzpatrick T. Li P. Zhou Y. Lin Y.-f.Y. Duan Q. Luo G. Fan S. Lu Y. Feng A. Zhan Y. Liang B. Cai W. Zhang L. Du X. Zou H. (2020b). Clinical characteristics of coronavirus disease 2019 (COVID-19) in China: A systematic review and meta-analysis. Journal of Infection, 80(6), 656–665. 10.1016/j.jinf.2020.03.041 PMC7151416 32283155

[bibr13-10998004211055866] Ghayda R. A. Lee J. Lee J. Y. Kim D. K. Lee K. H. Hong S. H. Han Y. J. Kim J. S. Yang J. W. Kronbichler A. Smith L. Koyanagi A. Jacob L. Shin J. I. (2020). Correlations of clinical and laboratory characteristics of COVID-19: A systematic review and meta-analysis. International Journal of Environmental Research and Public Health, 17(14), 5026. 10.3390/ijerph17145026 PMC7400047 32668763

[bibr14-10998004211055866] Grant M. C. Geoghegan L. Arbyn M. Mohammed Z. McGuinness L. Clarke E. L. Wade R. G. (2020). The prevalence of symptoms in 24,410 adults infected by the novel coronavirus (SARS-CoV-2; COVID-19): A systematic review and meta-analysis of 148 studies from 9 countries. PLoS One, 15(6), e0234765. 10.1371/journal.pone.0234765 32574165 PMC7310678

[bibr15-10998004211055866] Han Y. Liu Y. Zhou L. Chen E. Liu P. Pan X. Lu Y. (2020). Epidemiological assessment of imported coronavirus disease 2019 (COVID-19) cases in the most affected city outside of Hubei Province, Wenzhou, China. JAMA Network Open, 3(4), e206785. 10.1001/jamanetworkopen.2020.6785 32324236 PMC7180422

[bibr16-10998004211055866] Higgins J. P. T. Thompson S. G. Deeks J. J. Altman D. G. (2003). Measuring inconsistency in meta-analyses. British Medical Journal, 327(7414), 557–560. 10.1136/bmj.327.7414.557 12958120 PMC192859

[bibr17-10998004211055866] Ke C. Yu C. Yue D. Zeng X. Hu Z. Yang C. (2020). Clinical characteristics of confirmed and clinically diagnosed patients with 2019 novel coronavirus pneumonia: A single-center, retrospective, case-control study. Medicina Clínica, 155(8), 327–334. 10.1016/j.medcli.2020.06.055 PMC7386390 32782109

[bibr18-10998004211055866] Kuang Y. Zhang H. Zhou R. Lin S. Lin M. Wang J. Pang P. Ma L. Ji W. (2020). Epidemiological and clinical characteristics of 944 cases of 2019 novel coronavirus infection of non-COVID-19 Exporting city, Zhejiang, China. 10.2139/ssrn.3543604

[bibr19-10998004211055866] Li Q. Guan X. Wu P. Wang X. Zhou L. Tong Y. Ren R. Leung K. S. M. Lau E. H. Y. Wong J. Y. Xing X. Xiang N. Wu Y. Li C. Chen Q. Li D. Liu T. Zhao J. Liu M. Feng Z. (2020a). Early transmission dynamics in Wuhan, China, of novel coronavirus-infected pneumonia. New England Journal of Medicine, 382(13), 1199–1207. 10.1056/NEJMoa2001316 PMC7121484 31995857

[bibr20-10998004211055866] Li J. Huang D. Q. Zou B. Yang H. Hui W. Z. Rui F. Yee N. T. S. Liu C. Nerurkar S. N. Kai J. C. Y. Teng M. L. P. Li X. Zeng H. Borghi J. A. Henry L. Cheung R. Nguyen M. H. (2021). Epidemiology of COVID-19: A systematic review and meta-analysis of clinical characteristics, risk factors, and outcomes. Journal of Medical Virology, 93(3):1449–1458. 10.1002/jmv.26424 32790106 PMC7436673

[bibr21-10998004211055866] Li J. Zhang Y. Wang F. Liu B. Li H. Tang G. Chang Z. Liu A. Fu C. Gao J. Li J. (2020b). Sex differences in clinical findings among patients with coronavirus disease 2019 (COVID-19) and severe condition. MedRxiv. 10.1101/2020.02.27.20027524

[bibr22-10998004211055866] Li M. Katikireddi S. V. (2019). Urban-rural inequalities in suicide among elderly people in China: A systematic review and meta-analysis. International Journal for Equity in Health, 18(1), 2. 10.1186/s12939-018-0881-2 30606191 PMC6319001

[bibr23-10998004211055866] Liu L. Gao J.-Y. Hu W. Zhang X. Guo L. Liu C. Tang Y. Lang C. Mou F. Yi Z. Pei Q. Sun K. Xiang J. Xiao J. (2020). Clinical characteristics of 51 patients discharged from hospital with COVID-19 in Chongqing, China. MedRxiv. 10.1101/2020.02.20.20025536

[bibr24-10998004211055866] Neyeloff J. L. Fuchs S. C. Moreira L. B. (2012). Meta-analyses and Forest plots using a microsoft excel spreadsheet: Step-by-step guide focusing on descriptive data analysis. BMC Research Notes, 5(1), 52. 10.1186/1756-0500-5-52 22264277 PMC3296675

[bibr25-10998004211055866] Nie S. Zhao X. Zhao K. Zhang Z. Zhang Z. Zhang Z. (2020). Metabolic disturbances and inflammatory dysfunction predict severity of coronavirus disease 2019 (COVID-19): A retrospective study. MedRxiv. 10.1101/2020.03.24.20042283

[bibr26-10998004211055866] Patsopoulos N. A. Evangelou E. Ioannidis J. P. (2008). Sensitivity of between-study heterogeneity in meta-analysis: Proposed metrics and empirical evaluation. International Journal of Epidemiology, 37(5), 1148–1157. 10.1093/ije/dyn065 18424475 PMC6281381

[bibr27-10998004211055866] Peckham H. de Gruijter N. M. Raine C. Radziszewska A. Ciurtin C. Wedderburn L. R. Rosser E. C. Webb K. Deakin C. T. (2020). Male sex identified by global COVID-19 meta-analysis as a risk factor for death and ITU admission. Nature Communications, 11(1), 6317. 10.1038/s41467-020-19741-6 PMC7726563 33298944

[bibr28-10998004211055866] Qi S. Guo H. Shao H. Lan S. He Y. Tiheiran M. Li H. (2020a). Computed tomography findings and short-term follow-up with novel coronavirus pneumonia. MedRxiv. 10.1101/2020.04.02.20042614

[bibr29-10998004211055866] Qi D. Yan X. Tang X. Peng J. Yu Q. Feng L. Yuan G. Zhang A. Chen Y. Yuan J. Huang X. Zhang X. Hu P. Song Y. Qian C. Sun Q. Wang D. Tong J. Xiang J. (2020b). Epidemiological and clinical features of 2019-nCoV acute respiratory disease cases in Chongqing municipality, China: A retrospective, descriptive, multiple-center study. MedRxiv. 10.1101/2020.03.01.20029397

[bibr30-10998004211055866] Qin X. Qiu S. Yuan Y. Zong Y. Tuo Z. Li J. Liu J. (2020). Clinical characteristics and treatment of patients infected with COVID-19 in Shishou, China. 10.2139/ssrn.3541147

[bibr31-10998004211055866] Rodriguez-Morales A. J. Cardona-Ospina J. A. Gutiérrez-Ocampo E. Villamizar-Peña R. Holguin-Rivera Y. Escalera-Antezana J. P. Alvarado-Arnez L. E. Bonilla-Aldana D. K. Franco-Paredes C. Henao-Martinez A. F. Paniz-Mondolfi A. Lagos-Grisales G. J. Ramírez-Vallejo E. Suárez J. A. Zambrano L. I. Villamil-Gómez W. E. Balbin-Ramon G. J. Rabaan A. A. Harapan H. Sah (2020). Clinical, laboratory and imaging features of COVID-19: A systematic review and meta-analysis. Travel Medicine and Infectious Disease, 34, 101623. 10.1016/j.tmaid.2020.101623 32179124 PMC7102608

[bibr32-10998004211055866] Shi Q. Zhao K. Yu J. Jiang F. Feng J. Zhao K. Zhang X. Chen X. Hu P. Hong Y. Li M. Liu F. Chen C. Wang W. (2020). Clinical characteristics of 101 COVID-19 nonsurvivors in Wuhan, China: A retrospective study. MedRxiv. 10.1101/2020.03.04.20031039

[bibr33-10998004211055866] Song C.-Y. Xu J. He J.-Q. Lu Y.-Q. (2020). COVID-19 early warning score: A multi-parameter screening tool to identify highly suspected patients. MedRxiv. 10.1101/2020.03.05.20031906

[bibr34-10998004211055866] Sun P. Qie S. Liu Z. Ren J. Li K. Xi J. (2020). Clinical characteristics of hospitalized patients with SARS-CoV-2 infection: A single arm meta-analysis. Journal of Medical Virology, 92(6), 612–617. 10.1002/jmv.25735 32108351 PMC7228255

[bibr35-10998004211055866] Tao Y. Cheng P. Chen W. Wan P. Chen Y. Yuan G. Chen J. Huo D. Guan G. Sun D. Tan J. Yang G. Zeng W. Zhu C. (2020). High incidence of asymptomatic SARS-CoV-2 infection, Chongqing, China. MedRxiv. 10.1101/2020.03.16.20037259

[bibr36-10998004211055866] Wan S. Li M. Ye Z. Yang C. Cai Q. Duan S. Song B. (2020). CT manifestations and clinical characteristics of 1115 patients with coronavirus disease 2019 (COVID-19): A systematic review and meta-analysis. Academic Radiology, 27(7), 910–921. 10.1016/j.acra.2020.04.033 32505599 PMC7200137

[bibr37-10998004211055866] Well G. A. Shea B. O’Connell D. Peterson J. Welch V. Losos M. Tugwell P. (2020). The Newcastle-Ottawa Scale (NOS) for assessing the quality of nonrandomised studies in meta-analyses. http://www.ohri.ca/programs/clinical_epidemiology/oxford.asp

[bibr38-10998004211055866] Wen Y. Wei L. Li Y. Tang X. Feng S. Leung K. Wu X. Pan X.-F. Chen C. Xia J. Zou X. Feng T. Mei S. (2020). Epidemiological and clinical characteristics of COVID-19 in Shenzhen, the largest migrant city of China. MedRxiv. 10.1101/2020.03.22.20035246

[bibr39-10998004211055866] WHO . (2021). WHO coronavirus (COVID-19) dashboard. https://covid19.who.int

[bibr40-10998004211055866] Wiebers D. O. Feigin V. L. (2020). What the COVID-19 crisis is telling humanity. Neuroepidemiology, 54(4), 283–286. 10.1159/000508654 32498068 PMC7316645

[bibr41-10998004211055866] World Health Organization . (2020). Statement on the second meeting of the International Health Regulations (2005) Emergency Committee regarding the outbreak of novel coronavirus (2019-nCoV). https://www.who.int/news-room/detail/30-01-2020-statement-on-the-second-meeting-of-the-international-health-regulations-(2005)-emergency-committee-regarding-the-outbreak-of-novel-coronavirus-(2019-ncov)

[bibr42-10998004211055866] Xiao Y. Huang S. Yan L. Wang H. Wang F. Zhou T. Deng J. He M. (2020). Clinical characteristics of diarrhea in 90 cases with COVID-19: A descriptive study. International Emergency Nursing, 52, 100912. 10.1016/j.ienj.2020.100912 32827932 PMC7414355

[bibr43-10998004211055866] Xie J. Ding C. Li J. Wang Y. Guo H. Lu Z. Wang J. Zheng C. Jin T. Gao Y. He H. (2020). Characteristics of patients with coronavirus disease (COVID-19) confirmed using an IgM-IgG antibody test. Journal of Medical Virology, 92(10):2004–2010. 10.1002/jmv.25930 32330303 PMC7264659

[bibr44-10998004211055866] Xu Y. Li Y. Zeng Q. Lu Z. Li Y. Wu W. Dong S. Huang G. Wang X. (2020). Clinical characteristics of SARS-CoV-2 pneumonia compared to controls in Chinese Han population. MedRxiv. 10.1101/2020.03.08.20031658

[bibr45-10998004211055866] Yang J. Zheng Y. Gou X. Pu K. Chen Z. Guo Q. Ji R. Wang H. Wang Y. Zhou Y. (2020). Prevalence of comorbidities and its effects in patients infected with SARS-CoV-2: A systematic review and meta-analysis. International Journal of Infectious Diseases, 94, 91–95. 10.1016/j.ijid.2020.03.017 32173574 PMC7194638

[bibr46-10998004211055866] Yin Y. Wunderink R. G. (2018). MERS, SARS and other coronaviruses as causes of pneumonia. Respirology, 23(2), 130–137. 10.1111/resp.13196 29052924 PMC7169239

[bibr47-10998004211055866] Zhao W. Yu S. Zha X. Wang N. Pang Q. Li T. Li A. (2020). Clinical characteristics and durations of hospitalized patients with COVID-19 in Beijing: A retrospective cohort study. MedRxiv. 10.1101/2020.03.13.20035436

[bibr48-10998004211055866] Zhao Z. Zhang F. Xu M. Huang K. Zhong W. Cai W. Yin Z. Huang S. Deng Z. Wei M. Xiong J. Hawkey P. M. (2003). Description and clinical treatment of an early outbreak of severe acute respiratory syndrome (SARS) in Guangzhou, PR China. Journal of Medical Microbiology, 52(8), 715–720. 10.1099/jmm.0.05320-0 12867568

[bibr49-10998004211055866] Zheng F. Tang W. Li H. Huang Y.-X. Xie Y.-L. Zhou Z.-G. (2020). Clinical characteristics of 161 cases of corona virus disease 2019 (COVID-19) in Changsha. European Review for Medical and Pharmacological Sciences, 24(6), 3404–3410. 10.26355/eurrev_202003_20711 32271459

[bibr50-10998004211055866] Zhou Z. Zhao N. Shu Y. Han S. Chen B. Shu X. (2020). Effect of gastrointestinal symptoms in patients with COVID-19. Gastroenterology, 158(8), 2294–2297. 10.1053/j.gastro.2020.03.020 32199880 PMC7270807

[bibr51-10998004211055866] Zhu J. Ji P. Pang J. Zhong Z. Li H. He C. Zhang J. Zhao C. (2020). Clinical characteristics of 3062 COVID-19 patients: A meta-analysis. Journal of Medical Virology, 92(10), 1902–1914. 10.1002/jmv.25884 32293716 PMC7262119

