# Supplementary material for: Clinical Features of COVID-19 Patients in the First Year of Pandemic: A Systematic Review and Meta-Analysis
Source: Biol Res Nurs. 2021 Dec 4;24(2):172–85. doi: 10.1177/10998004211055866 (PMC8968436; doi:10.1177/10998004211055866)
Supplement: sj-pdf-1-brn-10.1177_10998004211055866 – Supplemental Material for Clinical Features of COVID-19 Patients in the First Year of Pandemic: A Systematic Review and Meta-Analysis [file sj-pdf-1-brn-10.1177_10998004211055866.pdf]

Supplementary Table 1. General Characteristics of the Studies

	Study	Country	Continent	Month	Preprint	Sample Size	Female	Age mean	Fever	Cough	Fatigue	Dyspnoea	Diarrhoea	Nausea & Vomiting	Headache	Expectorant	Hemoptysis	Anorexia	Sore throat	Chills	Myalgias	Rhinorrhea	Comorbidities	Hypertension	Diabetes	Cardiovascular	Respiratory	Renal	Liver	Malignancy	Cerebrovascular	Method of Diagnosis	NOS
1	(Zhu et al., 2020)	China	Asia	February	N	72	30	55.6	59	28	20	16	9																			RT-PCR	M
2	(L. Shi et al., 2020)	China	Asia	February	N	161	57	59.38	127	111	17	36	6	6	5	19								44	25	8	11			7	18	NG	M
3	(Jiangshan Lian et al., 2020)	China	Asia	February	N	788	381		636	506	186	37	88		75	265	15		111		91	47	218	126	57	11	3	7	31	6		RT-PCR	M
4	(Q. Yang et al., 2020)	China	Asia	February	N	136	70	56	125	117	53	19	22		12			73						36	20	9		4	8	4		RT-PCR	M
5	(X.-W. Xu et al., 2020)	China	Asia	January	N	62	27	41	48	50	32		3		21	35	2						20	5	1		1	1	1			RT-PCR	M
6	(S. Shi et al., 2020)	China	Asia	February	N	416	211	64	334	144	55	117	16		9	23			12		19	10		127	60	61	12	14	4	9	7	RT-PCR	M
7	(Tenforde et al., 2020)	USA	North America	May	N	316	185	43	167	182	198	114	144	128	171	91	16		89	156	167	110		97	51	19	65	14	11			NG	M
8	(Duanmu et al., 2020)	USA	North America	March	N	100	44	45	71	87	21	38	10	15	15				21		21	16		19	10		11	6	14		18	RT-PCR	M
9	(Bernheim et al., 2020)	China	Asia	February	N	121	60	45	74	58						20																RT-PCR	L
10	(Wei et al., 2020)	China	Asia	March	N	556	289	61.5	445	264	258	79			28								18	6	3			1		1		RT-PCR	M
11	(Suleyman et al., 2020)	USA	North America	April	N	463	259	57.5	315	347	194	282	100	147	74			100			194	113		295	178	108	122	208		49		RT-PCR	L
12	(TieLong Chen et al., 2020)	China	Asia	February	N	203	95	54	181	122	16	72	10	6	10			6			54		88	43	16	16	12	8	8	7		RT-PCR	L
13	(D'Silva et al., 2020)	USA	North America	April	N	156	108	62	100	111		70	44		37				51		66	41		84	42	37	49					RT-PCR	M
14	(Yuchen Chen et al., 2020)	China	Asia	March	N	904	483	56	576	443	212	122	47	20		94					117			273	136	91	22	42		19	52	RT-PCR	M
15	(Covino et al., 2020)	Italy	Europe	March	N	69	32	84	56	29	8	32												41	9	21	7			3	28	RT-PCR	M
16	(Q. Shi, Zhang, et al., 2020)	China	Asia	March	N	306	156	65	238	173	173	112	41	5	9			163			38			131		49	21	12	11	14		RT-PCR	M
17	(H. Xu et al., 2020)	China	Asia	March	N	102	52		75	72	6	11	11			34					15			18	18	9	5	6				RT-PCR	M
18	(S. Liu et al., 2020)	China	Asia	March	N	625	296	44	412	344						166								91	40						10	RT-PCR	M
19	(Tao Chen et al., 2020)	China	Asia	February	N	274	103	68	249	185	137	120	77	40	31	83	7	66			60	12		93	47	23	18	4	11	7		RT-PCR	M
20	(D. Wang, Hu, et al., 2020)	China	Asia	February	N	138	63	56	136	82	96	43	14	19	9	37		55			48		64	43	14	20	4	4	4	10		RT-PCR	L
21	(J.-J. Zhang, Dong, et al., 2020)	China	Asia	February	N	120	59	57	110	90	90	44	55	24				17					90	42	17	7	4	2	7		10	RT-PCR	M
22	(Q. Chen et al., 2020)	China	Asia	March	N	145	66	47.5	109	118	59	47	39	17	24			62			20	28		22	14	2		3	7	3		RT-PCR	M
23	(F. Zheng et al., 2020)	China	Asia	February	N	161	81	45	122	101	64	23	17		12								33	22	4	6	4	4	4			NG	M
24	(H. Zhang, Shang, et al., 2020)	China	Asia	February	N	194	86	48.3	146	86	44	71	36		33				98		44											RT-PCR	M

25	(T. Li, Lu, et al., 2020)	China	Asia	February	N	312	125	69.2	252	239	83	167	25		28	67				33	18		241	178	121	93	27	11	10	12		RT-PCR	L
26	(Colaneri et al., 2020)	Italy	Europe	February	N	44	16	67.5	40	15	2	10	3	22									28	15	7	11	2	2	2	6		RT-PCR	M
27	(Guan, Ni, et al., 2020)	China	Asia	January	N	1099	459	47	975	745	419	205	42	55	150	370	10		153	126	164	19	261	165	81	15	12	8	23	10		RT-PCR	L
28	(Goyal et al., 2020)	USA	North America	April	N	393	155	62.2	303	312		222	93	75							107			197	99	82	69	18	6	23		RT-PCR	L
29	(F. Wu et al., 2020)	China	Asia	February	N	998	449	56	869	694	401	262	36	58	122	347	12		99	109	158		50	19	8	18			1	1		RT-PCR	M
30	(Xiao et al., 2020)	China	Asia	February	N	90	39	61	80	62	43	28	8	37	16		2	22	4		19		57	35	18	15	7		8			NG	M
31	(Guo et al., 2020)	China	Asia	February	N	105	57	67	70	68	35	31	10	6	9			9			8		73	46	27	17	9	5	5			RT-PCR	M
32	(Y. Deng et al., 2020)	China	Asia	February	N	225	101		189	85	57	99	33		13	49	7					4	127	58	26	17	25			8		RT-PCR	L
33	(J. Wu et al., 2020)	China	Asia	February	N	80	41	46	63	51	18	30	1	1	13						18	5	38		5	25	3	1	1	1		RT-PCR	M
34	(Kui Liu et al., 2020)	China	Asia	January	N	137	76	57	112	66	44	26	11		13	6	7						27	13	14	10	2			2		RT-PCR	M
35	(Niu et al., 2020)	China	Asia	February	N	141	71		110	71	40	23			11								26	15	3	5	9				2	RT-PCR	M
36	(X.-Y. Zhao et al., 2020)	China	Asia	February	N	91	42	46	75	59	35	21	14					11		21	15	19	21	18	3	1	1	1		3		RT-PCR	M
37	(B. Y. Yang et al., 2020)	USA	North America	March	N	124	66	75.7	68	43	59	64	9	14	4				3		1			44	25	78	26	7		5	46	RT-PCR	M
38	(Yafei Wang, Zhou, et al., 2020)	China	Asia	February	N	110	62		91	68	41	37												23	15		6				7	RT-PCR	M
39	(Mo et al., 2020)	China	Asia	February	N	155	69	54	126	97	60	61	7	6	8			26			50			37	15	15	8	6	7	7	7	RT-PCR	M
40	(Yang Xu et al., 2020)	China	Asia	February	N	69	34	57	68	43	40	11	7	6	23	27		8	10	10												RT-PCR	M
41	(Yu, Lei, Li, Wang, Liu, et al., 2020)	China	Asia	March	N	1464	728	64	1259	520	346	69	74	34	29			63			54	23		306	211	117	50	27	36	17	47	RT-PCR	L
42	(D. Wang, Yin, et al., 2020)	China	Asia	February	N	107	50	51	104	67	69	35	7	9	7			33			33	12	41	26	11	13	3	3	6		6	RT-PCR	M
43	(X. Yang et al., 2020)	China	Asia	February	N	52	17	59.7	51	40		33	2	2	3						6		21		9	5	4			2	7	RT-PCR	M
44	(F. Zhou et al., 2020)	China	Asia	February	N	191	72	56	180	151	44	56	9			44					29		91	58	36	15	6	2		2		RT-PCR	L
45	(T. Xu et al., 2020)	China	Asia	February	N	51	26		34	23	2	4	5			13					8	3	12		4	5	1	1	1			RT-PCR	M
46	(Guqin Zhang, Hu, et al., 2020)	China	Asia	February	N	221	113	55	200	136	156	64	25		17			80				22	78	54	22	2	6	6	7	9	15	RT-PCR	M
47	(J. Cao et al., 2020)	China	Asia	February	N	102	49	54	83	50	56		11								35		47	28	11	5	10	4	2	4	6	RT-PCR	M
48	(Wan et al., 2020)	China	Asia	February	N	135	63	47	120	102	44	18	18		34	12	4	6		14		24	43	13	12	7	1		2	4		RT-PCR	M
49	(Z. Wang, Yang, et al., 2020)	China	Asia	February	N	69	37	42	60	38	29	20	10	3	10	20		7			21	6		9	7	8	6		1	4		RT-PCR	M
50	(Kai Liu et al., 2020)	China	Asia	February	N	56	25		44	21	5	4												10	4	3		1				RT-PCR	M
51	(C. Huang et al., 2020)	China	Asia	February	N	41	11	49	40	31	18	22	1		3	11	2						13	6	8	6	1		1	1		RT-PCR	M
52	(Yonghao Xu et al., 2020)	China	Asia	February	Y	45	16	56.7	39	32		29									4		26	21	13	6	4			3		RT-PCR	M
53	(Jun Chen et al., 2020)	China	Asia	February	N	249	123	51	217	91	39	19	8		28			8	16			17	90		25	55	5		2	1		RT-PCR	L
54	(Xiao Li, Fang, et al., 2020)	China	Asia	February	N	154	78	59	140	126																						RT-PCR	M
55	(S. Qi et al., 2020)	China	Asia	February	Y	57	26	46.5	48	28	18	7									10											RT-PCR	M
56	(L. Wang,	China	Asia	Februa	N	339	173	71	311	179	135	138	43		12	93		94			16			138	54	53	21	13	2	15	21	RT-	L

	He, et al., 2020)			ry																												PCR	
57	(C.-Y. Song et al., 2020)	China	Asia	February	Y	73	27	53	69	44	24	16	3		9	17				26		25		22	4	4	2	1				RT-PCR	M
58	(Ticinesi et al., 2020)	Italy	Europe	April	N	852	401	73	695	375	80	467												498	179	236	102	75		130	121	RT-PCR	M
59	(C. Qin, Zhou, Hu, Zhang, et al., 2020)	China	Asia	February	N	452	217	58	423	152	212	232	122	42	52	189	12	96			98	8	201	135	75	27	21	10	6	14	11	RT-PCR	L
60	(F. Song et al., 2020)	China	Asia	January	N	51	26		49	24	16	7	5	3		10		9	3			2	11	5	3	1	1		1			RT-PCR	M
61	(N. Chen et al., 2020)	China	Asia	January	N	99	32	55.5	82	81	11	31	2	1	8				5		11	4	50		13	40	1			1	1	RT-PCR	M
62	(P. Chen et al., 2020)	China	Asia	January	N	136	67	47	64	69	19	13	12		12																	RT-PCR	M
63	(Shahriarirad et al., 2020)	Iran	Europe	March	N	113	42	53.75	67	73	75	58	25	77	60	24	7	75	36	67	67	26		22	16	16	16	6		1		RT-PCR	M
64	(D. Qi et al., 2020)	China	Asia	February	Y	267	118	48	225	189	208	43	10	10				46		30	136	6	41	20	26	13	25					RT-PCR	M
65	(Han et al., 2020)	China	Asia	March	N	482	147	48	317	232	59	13	27		35				50	31	15	14										NG	M
66	(Jin et al., 2020)	China	Asia	February	N	651	320	46.14	545	435	119	27	74		67		11		99		71	37	178	100	48	5	1	6	25	6		RT-PCR	L
67	(X. Zhang, Cai, et al., 2020)	China	Asia	February	N	645	317		540	425	118	26	53	22	67	225	11		97				177	100	48	5	1	6	25	6		RT-PCR	L
68	(García-Azorín et al., 2020)	Spain	Europe	February	N	104	66	56.7	93	89	23	52	49		27	16		67			44	2		36	12	9	24			13		RT-PCR	M
69	(Tao et al., 2020)	China	Asia	March	Y	101			71	81	26	19														1						RT-PCR	M
70	(Jitian Li, Chen, et al., 2020)	China	Asia	February	N	655	288		580	276	184	33	33	41	80	169			70		78	41	150			89						RT-PCR	M
71	(Guoxin Zhang, Nie, et al., 2020)	China	Asia	February	N	112	79	38.6	61	52	29		11	4	4						2											RT-PCR	M
72	(Nie et al., 2020)	China	Asia	February	Y	97	63	39	57	54	32	8	12	12	7	15			10					15	5	7	2	3	3	3	3	RT-PCR	M
73	(Imam et al., 2020)	USA	North America	March	N	1305	603	61	852	921	471	827	246	385	115	96	79	215	78		295	246	947	734	393	208	405	303	25	83	108	RT-PCR	L
74	(Kluytmans-van den Bergh et al., 2020)	Netherlands	Europe	March	N	86	71	49	46	66	65	33	16		49			15	34		54	46										RT-PCR	M
75	(Wei Zhao et al., 2020)	China	Asia	February	N	101	45	44.4	79	63	17	1	3	2					12					3		16	5					RT-PCR	M
76	(C. Wu et al., 2020)	China	Asia	February	N	201	73	51	188	163	65	80				83								39	22	8	5	2	2	1	7	RT-PCR	M
77	(Chang et al., 2020)	Korea	Asia	March	N	211	137	37		92		26	33		53				46	22	44	58								1		RT-PCR	M
78	(H. Sun et al., 2020)	China	Asia	March	N	244	111		211	179			72											138	51	35	24					RT-PCR	M
79	(S. M. Shi, Bakaev, et al., 2020)	USA	North America	March	N	146	80	85	31	35			9	13				26														RT-PCR	M
80	(D. J. Lee et al., 2020)	Canada	North America	April	N	56	33	38	26	37	4	22	20		10				21			20			4	6	10					NG	M
81	(Easom et al., 2020)	UK	Europe	February	N	68	36	42.5	27	53	11	17		2					39			20										RT-PCR	M
82	(Alvarado et al., 2020)	USA	North America	April	N	736	164	25	55	332	146	23	65		252				195	52	386											RT-PCR	L
83	(X. Zhao et al., 2020)	China	Asia	February	N	80	37	44	71	40																						RT-PCR	M
84	(K. Li, Wu, et al., 2020)	China	Asia	February	N	83	39	45.5	72	65		9			9	15			6		15		15	5	7	1	5					RT-PCR	M

85	(X. Wang et al., 2020)	China	Asia	February	N	1012	488	50	761	531		231	152	36	152	220			144	182		57	114	46	27	15	20					RT-PCR	L
86	(Javanian et al., 2020)	Iran	Asia	March	N	100	49	60.12	77	82	77	82	14	45	59	45	7	86			50			32	37	20	12	12	3	4	3	RT-PCR	M
87	(G. -u Kim et al., 2020)	Korea	Asia	March	N	172	106	26	20	69	46	7	27	9	54		68	23	31	31	54	45										RT-PCR	M
88	(Berenguer et al., 2020)	Spain	Europe	March	N	4035	1602	70	3240	2862	2505	1953	471	389	431	956	99							2052	871	932	1014	199	54	359	688	RT-PCR	L
89	(Maechler et al., 2020)	Germany	Europe	April	N	333	144	34	121	218	212	61	51		187				159	122	17	162			7	15	30					RT-PCR	M
90	(Koleilat et al., 2020)	USA	North America	April	N	135	63		98	87	45		28	16							38			94	51	44	13	28	50	20		RT-PCR	M
91	(K. Wang, Kang, et al., 2020)	China	Asia	February	N	114	56	53	107	91		27	3			9			6				60	33	15	7	5			1	1	RT-PCR	M
92	(W. Wang, Xin, et al., 2020)	China	Asia	March	N	421	207	52	255	219		41	11		44	32			102	97	96		88	44	13		22			4	8	RT-PCR	M
93	(M. Huang et al., 2020)	China	Asia	March	N	60	25	57	48	31	4	7	4		4	3							31	14	10	3	1	1				RT-PCR	M
94	(Junli Li, Xu, et al., 2020)	China	Asia	February	N	74	30	66	67	34	49	49	6	2		13	6	41					56	35	14	6	6		2	2		RT-PCR	M
95	(M. Wang, Zhang, et al., 2020)	China	Asia	February	N	843	443	60	660	513	281	218	92	33	25	160			33		50	14		231	98	48	24	15	22	16	17	RT-PCR	L
96	(Ke et al., 2020)	China	Asia	March	N	194	79	64	139	31	12		12										135	73	39	18	11	13		13		RT-PCR + SARS-CoV-2 antibody	M
97	(Jinpeng Li et al., 2020)	China	Asia	February	N	54	45	46	54	44	48	11	4	96	4																	RT-PCR	M
98	(Xiaochen Li, Xu, et al., 2020)	China	Asia	February	N	548	269	60	476	415	258	310	179	45	62				28		111			166	83	34	31	10	5	24		RT-PCR	M
99	(Brendish et al., 2020)	UK	Europe	February	N	352	150	68	112	128	117	130	57		73	53		112	50	84	62	39		144	91	125	86	41	17	18	47	RT-PCR	M
100	(Q. Deng et al., 2020)	China	Asia	February	N	112	55	65	98	79		63												36	19	19	4					RT-PCR	M
101	(Akbariqomi et al., 2020)	Iran	Asia	April	N	595	194	55	419	368	332	363	116	188	207	107	43		81	317	320	61		172	148	112	87	58	32	12	13	RT-PCR	M
102	(Vandercam et al., 2020)	Belgium	Europe	May	N	176	132	40.8	121	133	93	66	54		132						115		13	3	3							RT-PCR	M
103	(Fang-fang Chen et al., 2020)	China	Asia	April	N	681	319	65	584	462	352	123	119											293	114	80	15	27			33	RT-PCR	L
104	(Ying Sun et al., 2020)	China	Asia	April	N	63	26	47	53	34		11	5		8	19		17	5	1	16	1	29	13	5	2	2		2	1	2	RT-PCR	M
105	(R. Wang, Pan, et al., 2020)	China	Asia	February	N	125	54	38.7	116	102	43	57	50	24	11	52	4				4	4	34		10	18	2		1	1	1	RT-PCR	M
106	(Xudan Chen et al., 2020)	China	Asia	March	N	267	146	49	179	156	60	29	19	21	31	74		47	34		46	33	105	57	23	20	9		10	5		RT-PCR	M
107	(J. yeon Lee et al., 2020)	Korea	Asia	April	N	694	482	52.1	111	328		91	166		140	294				126	115	162		131	81	27	20	5	5	23	27	RT-PCR	L
108	(Y. Zheng et al., 2020)	China	Asia	February	N	99	48	49.4	85	84	72	35	2				12				12		41	21	6							RT-PCR	M
109	(Tian et al., 2020)	China	Asia	March	N	751	379	64	514	368	153	152	89	34	37	135			46	42				292	198	74	4	23	10	232	23	RT-PCR	M
110	(K. Yang et al., 2020)	China	Asia	March	N	205	109	63	159	151	75	71	24	14		70				17				67	22	16	5	4		205		RT-PCR	M
111	(Akter et al., 2020)	Bangladesh	Asia	June	N	734	176	39	554	421		195	149	61					175	100	294	147			146	67	45		16	10		RT-PCR	M
112	(Yu, Lei, Li, Wang, Li, et al., 2020)	China	Asia	February	N	1663	825	64	1427	598	392	198	76	36	29			69			57	4		347	245	131	62	31	38	19	57	RT-PCR	M
113	(Al-Omari et al., 2020)	KSA	Asia	May	N	401	80		145	215	106	88	31		65	31	3	13	88		58	29		60	41	11	15	1	19			RT-PCR	M

114	(Samrah et al., 2020)	Jordan	Asia	April	N	81	44	39	14	31	15	9	3		14		1			9			25	17	10	6			7	1		RT-PCR	M
115	(Almazeedi et al., 2020)	Kuwait	Asia	April	N	1096	208	41	931	314	38	30	16	18	70	24	1		129	308	75		335	177	155	41	48	11	71	14	7	RT-PCR	L
116	(Ruan et al., 2020)	China	Asia	February	N	150	48		127	110	37	110				54	3				19		77	52	25	13	4	2	4	3		RT-PCR	M
117	(Peng et al., 2020)	China	Asia	February	N	112	59	62	101	76	71	38	15									10	112	92	62							RT-PCR	M
118	(S. Wang, Chen, et al., 2020)	China	Asia	February	N	165	73	44	126	99	49	17	14				3	5		18		9	64	24	12	8	11	4	10	7		RT-PCR	M
119	(Nouri-Vaskeh et al., 2020)	Iran	Asia	May	N	111	33	73	37	65		80	5	5	5	8			2	24	25			50	41	24	26	11	4	7	13	RT-PCR	M
120	(Khan et al., 2020)	Pakistan	Asia	April	N	121	36	47	88	72		69	21	15	38				44		57			15	13	9	7		3	1		RT-PCR	M
121	(L. Liu et al., 2020)	China	Asia	February	Y	51	19	45	43	38	22	11	4	6	5	16					6	3		4	4				1			RT-PCR	M
122	(Jing Li, Zhang, et al., 2020)	China	Asia	February	Y	47	19	62	34	36	7	3	3		3	2			1		5		30	17	7	7	7		4	7		RT-PCR	M
123	(Z. Chen et al., 2020)	China	Asia	February	Y	89	59	33.3	40	40	13	7	5			10					10											RT-PCR	M
124	(Q. Shi, Zhao, et al., 2020)	China	Asia	February	Y	101	41	71	82	56	36	59	10	3	5	21	2	7			6			59	22	24	14	11		7	13	RT-PCR	M
125	(J. Liu et al., 2020)	China	Asia	February	N	64	41	35	43	30	22	10	3		8	8		4	16	3	14		8	3	2						1	RT-PCR	M
126	(Jingli Chen et al., 2020)	China	Asia	February	N	62	21	72	52	41	15		12	8	11	21		23			23		45		13	33	5			2		RT-PCR	M
127	(T. Yao et al., 2020)	China	Asia	February	N	83	30	71.8	78	50	75	81	4	2	3		5	70					66	47	14	26	16	5	3	5	14	RT-PCR	M
128	(L. Chen et al., 2020)	China	Asia	February	N	534	266	45	327	348	210	120	168		121			146	109		155			70	38	18	37	3	25	3		RT-PCR	M
129	(Wen Zhao et al., 2020)	China	Asia	February	Y	77	43	52	66	24	21	16	1	9	10	25			5		9	8	24	16	6	9	6	5		4	2	RT-PCR	M
130	(Wen et al., 2020)	China	Asia	February	Y	417	220	45.4	281	143		31	29		55	63			62		119	36										RT-PCR	M
131	(Fu et al., 2020)	China	Asia	February	Y	50	23	64	28	24	39	50	11	7		10		33	1		6			10	12	11	3	1	2			RT-PCR	M
132	(P. Shi, Ren, et al., 2020)	China	Asia	February	Y	134	69	46	87	96	55	12	13	14	12	61		45	21		13	7		20	9	6	5		5	5	6	RT-PCR	M
133	(Alshukry et al., 2020)	Kuwait	Asia	April	N	171	64	44.6	94	89	20	14	9	10	24	21			51	7	37	13		50	32	18	12	8		4		RT-PCR	M
134	(Duan et al., 2020)	China	Asia	February	Y	616	262	64	425	375	211	214	82						68		111		276	188	78	56	14	7	9		9	RT-PCR	M
135	(Ayed et al., 2020)	Kuwait	Asia	April	N	103	15	53	61	48	15		1	15					32					36	40	12	13	4	11	3		RT-PCR	M
136	(Nagura-Ikeda et al., 2020)	Japan	Asia	May	N	130	53	45	119	69		15	31		39								66	23	5	9	23			3		RT-PCR	M
137	(Y. Zhao et al., 2020)	USA	North America	April	N	722	272	63	520	487		507	121											373	210	52	128	46	11	112		RT-PCR	L
138	(X. Yan et al., 2020)	China	Asia	February	N	218	96	42.9	145	162	77	42	16		28	99			25	39	41	6			27	38	14	4	13	2	6	RT-PCR	M
139	(G. Li, Deng, et al., 2020)	China	Asia	March	N	199	110	63	148	133	50	53	6	28	20			32							76							RT-PCR	M
140	(Guan, Liang, et al., 2020)	China	Asia	January	N	1590	686	48.9	1351	1052	584	331	57	80	205	513	16		194	163	234		399	269	30	59	24	21	28	18	30	RT-PCR	L
141	(Cai et al., 2020)	China	Asia	March	N	149	70	41	96	68	22								16				46	24	8	3	3		5			RT-PCR	M
142	(Y. Xie et al., 2020)	China	Asia	March	N	62	35	66	46	29	16	11	9		3	12				7	7			24	13	32						RT-PCR	M
143	(M. Zhao et al., 2020)	China	Asia	March	N	1000	534	61	754	597	335	255	100	47	32	190					66	20	595	282	118	60	35	24	29	28	32	RT-PCR	L
144	(Lai et al., 2020)	China	Asia	March	N	57	35	52	47	31	9	2	5		6	16		2					10	5	2	1	2		2	1	1	RT-PCR	M

145	(Favà et al., 2020)	Spain	Europe	March	N	104	44	60	81	71		38	32								34		104	90	32	31	16	104		8		RT-PCR	M
146	(J.-J. Zhang, Cao, et al., 2020)	China	Asia	March	N	289	135	57	241	188	175	101	29					48			41		169	81	27	18	7	2				RT-PCR	M
147	(W. Shi, Gao, et al., 2020)	China	Asia	March	N	65	35		46	36	7	27	2	5	1	9						1										RT-PCR	M
148	(Z. Huang et al., 2020)	China	Asia	March	N	50	23		47	33	18	12	6			10		9			6				4	1	1					RT-PCR	M
149	(Bergquist et al., 2020)	USA	North America	May	N	208	144	47.8	35	157	113	78	78		129				73	94	58	104	145	78		10	51	8				RT-PCR	M
150	(Jalili et al., 2020)	Iran	Asia	April	N	28981	12620	57.3	13107	15144		13081					11986				6026		6666		3098	3288	1256	584	118	370		RT-PCR	L
151	(Xiaoping Chen et al., 2020)	China	Asia	March	N	123	50	51	85	62	67	26	20		21						40		35	19	12	8	5		18	5		RT-PCR	M
152	(Yinxiaohe Sun et al., 2020)	Singapore	Asia	February	N	54	25	42	54	36		7	20			13			18			12	5		5							RT-PCR	M
153	(Jing Chen et al., 2020)	China	Asia	March	N	3309	1667	62	2275	1868	490	1049	620			1381				295	237			988	464	242		57		93		RT-PCR	L
154	(Perez-Guzman et al., 2020)	UK	Europe	April	N	614	232	69	501	453		400	192										383	227	170	61	132	106	53	52	44	RT-PCR	M
155	(X. Liu et al., 2020)	China	Asia	March	N	58	27	29	29	24	6	2	4		6							11		7	4	3	4					RT-PCR	L
156	(Yuhong Chen et al., 2020)	China	Asia	March	N	51	24	58	48	34	14	18	6		4	19							33	21	11	12	8	2	3	1	5	RT-PCR	M
157	(Sepulchre et al., 2020)	Belgium	Europe	March	N	198	81	62	147	124		150									28		92	82	47	41	54	55			41	RT-PCR	M
158	(Fuyang Chen et al., 2020)	China	Asia	February	N	660	365	55	524	431	237		53		42						155		326	230	114	67	43	36				NG	M
159	(S.-P. Dai et al., 2020)	China	Asia	February	N	68	33		56	48	4	4				13				4			42	14	11	22	5	4	7			RT-PCR	M
160	(M. K. Kim et al., 2020)	Korea	Asia	March	N	1082	700	56.5	466	530		268	197		286	406			203		326	156	496	374	235	74	72	32		50	66	RT-PCR	L
161	(Y. Zhang, Li, et al., 2020)	China	Asia	April	N	166	81	62.7	139	136	99	115	77		53	90	16	75	30		63			76	61	12	19	9	5	3	12	RT-PCR	M
162	(Y. Yan et al., 2020)	China	Asia	February	N	193	79	64	173	135	101	115	51	19	21			68					94	73	48	31	14	4	1		8	RT-PCR	M
163	(X. Yao et al., 2020)	China	Asia	March	N	2474	1239	61	1818	1680	511	540			545									745	355	202					143	RT-PCR	L
164	(Jing Li, Ding, et al., 2020)	China	Asia	February	N	74	39	44.26	62	58		12				39								11	7	1	2			1		RT-PCR	M
165	(J. Zhou et al., 2020)	China	Asia	February	N	201	99	45	135	118	65	16	17	8	18						21	10				41						RT-PCR	M
166	(Jishou Zhang, Wang, et al., 2020)	China	Asia	February	N	869	492	51	565	424	226	47	58	36	18	36			18		114	14	121	91	21	5	11	4	5	6	1	RT-PCR	L
167	(Steinmeyer et al., 2020)	France	Europe	May	N	93	52	85.5	56	36	64	52	12	8	4	18				10	10			65	11	46	35				43	RT-PCR	M
168	(Zerah et al., 2020)	France	Europe	May	N	821	473	86	452	367	518	368	120	61	33						97			547	204	247	96	297	82	87	443	RT-PCR	L
169	(Ramos-Rincon et al., 2020)	Spain	Europe	February	N	2772	1405	86.3	590	1694	1026	1705	396	150										2080	710	855	547	324	1399	364	952	RT-PCR	L
170	(Qian et al., 2020)	China	Asia	February	N	91	54	50	65	55	40	3	21	17	7	30	10	23			5			15	8	3						RT-PCR	M
171	(H. Dai et al., 2020)	China	Asia	February	N	234	98	44.6	170	150	31	8	9	5					35	23	22	14										RT-PCR	M
172	(G. Yang et al., 2020)	China	Asia	February	N	462	253		330	300			72		60									126	72	36	22	7	26		18	RT-PCR	M
173	(Mohamud et al., 2020)	Somalia	Africa	April	N	60	18	45.7	43	45	32	10	10	10	10				15					3	7		1					RT-PCR	M

174	(Khraise et al., 2020)	Jordan	Asia	May	N	108	63	36.4	16	43		14			14					10				19	11	5			7	2		RT-PCR	M
175	(Peng Yudong et al., 2020)	China	Asia	February	N	244	131	61	197	164	155	29	30									23		202	53	134						RT-PCR	M
176	(Myers et al., 2020)	USA	North America	May	N	377	165	61	127	120	185													164	118	22	28	48	21	18		RT-PCR	M
177	(Barry et al., 2020)	KSA	Asia	May	N	99	33	44	69	61	26	43	19		11								53	22	31	12	7	2	6			RT-PCR	M
178	(Almalki et al., 2020)	KSA	Asia	May	N	458	60	38.8	324	277	29	128	44	30					62		7	17		50	62	10	16	9	4	2	8	RT-PCR	M
179	(Chu et al., 2020)	China	Asia	February	N	54	18	39	36	17	9	5	3		4	3		3	1	2	3	1										RT-PCR	M
180	(J. Xie et al., 2020)	China	Asia	February	N	56	32	56.5	42	23	16	20	9						7	3	4		14	7	3				3	1		RT-PCR & IgM-IgG antibody	M
181	(Gayam et al., 2020)	USA	North America	May	N	408	177	67	219	255	143	273	111								179			271	176	99	97	69	66		99	RT-PCR	M
182	(Shu et al., 2020)	China	Asia	February	N	571	293	50	299	402	95	105	51	21	55			28				17		55	17	12		1				RT-PCR	M
183	(Lechien et al., 2020)	Belgium	Europe	April	N	1420	962	39.17	645	897	514	274	473	272	998	193		649	751		887	854		131	24	25	10	6	8	22	13	RT-PCR	L
184	(Z. Cao et al., 2020)	China	Asia	February	N	80	42	53	69	57	30	30	5	6	8	34		24		8	12			20	6	10	5					RT-PCR	M
185	(Lapostolle et al., 2020)	France	Europe	April	N	1487	752	44	1344	1368	864	463	352	456	803		39	305	377		823	207										RT-PCR	M
186	(R. Li, Tian, et al., 2020)	China	Asia	February	N	225	105	50	190	127		9												47								RT-PCR	M
187	(Casas-Rojo et al., 2020)	Spain	Europe	June	N	15111	6468	69.4	12723	8751	6507	8684	3554			2331							13358	7689	2924	3001	2071	917	5990	1610		RT-PCR	L
188	(Z. Zhou et al., 2020)	China	Asia	February	N	254	139	50.6	213	98	133	10	46	36	28	107			16		86			63	26	17	6		3	2	13	NG	M
189	(X. Qi et al., 2020)	China	Asia	March	N	70	31		53	53			7								12		11	8	3	2	1			2		RT-PCR	M
190	(Medetalibeyoglu et al., 2020)	Turkey	Europe	May	N	362	138	56	265	304	328	158	47			10							210	138		56	41	8	4	27	7	RT-PCR	M
191	(J. Song et al., 2020)	China	Asia	March	N	69	41	52	50	36	12	17	8		7	7	7	5	6		7	5	24	16	9	5	1			2	2	RT-PCR	M
192	(A. Wang, Gao, et al., 2020)	China	Asia	March	N	130	54	46.5	114	83			10			42						8	36	22	12	4	4	2	5		2	RT-PCR	M
193	(Y. He et al., 2020)	China	Asia	April	N	336	135	65	298	255	161	203	93	31		97								128	78	59	28	8	3	14	15	RT-PCR	M
194	(Killerby et al., 2020)	USA	North America	April	N	531	303		428	455		284	225		206				167		253		318	243	111	20	101	30	9	34		RT-PCR	M
195	(Price-Haywood et al., 2020)	USA	North America	May	N	3481	2087		1185	705		343	63								59			1074	566	267	221	278	59	158		RT-PCR	L
196	(Nowak et al., 2020)	Poland	Europe	April	N	169	82	63.7	74	56	57	61	8	6										80	32	52	22	35		35	58	RT-PCR	M
197	(Izquierdo et al., 2020)	Spain	Europe	March	N	10504	4985	58.2	4904	5243		3294	1099		764	1934			127		919			3527	1646	5058	888	764	111			RT-PCR	L
198	(Vena et al., 2020)	Italy	Europe	March	N	317	104	71	285	156	57	167	18	14	14	9					18		207	149	49	63	18	22		23	28	RT-PCR	M
199	(W. Yang et al., 2020)	China	Asia	February	N	149	68	45.11	114	87	5	2	11	2	13	48			21	21		5			9	28	1			2		RT-PCR	M
200	(Tomlins et al., 2020)	UK	Europe	April	N	95	35	75	68	70		41	11	13	9				6		13			35	37	54	31	22		20	14	RT-PCR	M
201	(Du et al., 2020)	China	Asia	February	N	85	23	65.8	78	19	50	60	16	4	4	32		48			14	2	58	32	19	17	2	3	5	6	7	RT-PCR	M
202	(Yang Wang et al., 2020)	China	Asia	February	N	344	165	64	301	233	167	208	92			135		91						141	64	40	16					RT-PCR	M
20	(Jie Zhang,	China	Asia	Februa	N	108	48	66	93	78	28	65	40	40	20	44					15			44	18	16	5	3	5	4	3	RT-	M

3	Meng, et al., 2020)			ry																												PCR	
204	(Feng et al., 2020)	China	Asia	February	N	141	69	44	105	74	31	5	6			16		5					33	21	8	3	4		4		1	RT-PCR	M
205	(S. He et al., 2020)	China	Asia	February	N	267	151	57	212	192	93	102	20		18						59			89	45	28	20	5		7	16	RT-PCR	M
206	(Ji et al., 2020)	China	Asia	February	N	49	18	43.6	39	17	9	6	4		9	11					6		16									RT-PCR	M
207	(X. Qin et al., 2020)	China	Asia	February	Y	89	43	55	86	83	30	27	3		15								25	15	10	3	5	1	5	2		RT-PCR	M
208	(Kuang et al., 2020)	China	Asia	February	Y	944	476	47.4	584	311	93				75				64		45											RT-PCR	M
209	(M. Chen et al., 2020)	China	Asia	February	Y	123	62		98	89	85	46			11		2							26	14	20	6	7		10		RT-PCR	M
210	(Yi Wang, Yao, et al., 2020)	China	Asia	February	Y	67	26	44	61	51	41	13	10								7		15	4	4		1	1	3	2		RT-PCR	M
211	(C. Qin, Zhou, Hu, Yang, et al., 2020)	China	Asia	February	N	1875	930	63	1469	741	477	834	311	139	123	514	21	449			259	29		641	295	189	55	41	24	54		RT-PCR	L
212	(Popov et al., 2020)	Bulgaria	Europe	June	N	138	51	52.9	109	95	124	39	7		95				46		67			69	12	22	6	7		10	3	RT-PCR	M
213	(Zhong et al., 2020)	China	Asia	March	Y	48	17	44.35	43	41	22	9	7	4	5		4	13	9	22	5		14	4	3	3			7			RT-PCR	M
214	(Xiong et al., 2020)	China	Asia	March	N	116	36	58.5	99	61	60	52	17	15	6	31	1	50		24	32	6	59	45	19	17	1		2	4	8	RT-PCR	M
215	(Jiang-shan Lian et al., 2020)	China	Asia	February	N	788	381		636	506	139	37	88		75	265	15		111		91		218	126	57	11	9	7	31	6		RT-PCR	M
	Total					132647	61352		84823	73778	28306	46681	14008	4093	9164	14159	12578	4126	5389	2910	16762	3452	29976	32925	18372	19086	9475	5434	8966	4863	3685		

References

Akbariqomi, M., Hosseini, M. S., Rashidiani, J., Sedighian, H., Biganeh, H., Heidari, R., … Kooshki, H. (2020). Clinical characteristics and outcome of hospitalized COVID-19 patients with diabetes: A single-center, retrospective study in Iran. *Diabetes Research and Clinical Practice*, *169*, 108467. https://doi.org/10.1016/j.diabres.2020.108467

Akter, F., Mannan, A., Mehedi, H. M. H., Rob, Md. A., Ahmed, S., Salauddin, A., … Hasan, Md. M. (2020). Clinical characteristics and short term outcomes after recovery from COVID-19 in patients with and without diabetes in Bangladesh. *Diabetes & Metabolic Syndrome*, *14*(6), 2031–2038. https://doi.org/10.1016/j.dsx.2020.10.016

Almalki, Z. S., Khan, M. F., Almazrou, S., Alanazi, A. S., Iqbal, M. S., Alqahtani, A., … Alahmari, A. K. (2020). Clinical Characteristics and Outcomes Among COVID-19 Hospitalized Patients with Chronic Conditions: A Retrospective Single-Center Study. *Journal of Multidisciplinary Healthcare*, *13*, 1089–1097. https://doi.org/10.2147/JMDH.S273918

Almazeedi, S., Al-Youha, S., Jamal, M. H., Al-Haddad, M., Al-Muhaini, A., Al-Ghimlas, F., & Al-Sabah, S. (2020). Characteristics, risk factors and outcomes among the first consecutive 1096 patients diagnosed with COVID-19 in Kuwait. *EClinicalMedicine*, *24*. https://doi.org/10.1016/j.eclinm.2020.100448

Al-Omari, A., Alhuqbani, W. N., Zaidi, A. R. Z., Al-Subaie, M. F., AlHindi, A. M., Abogosh, A. K., … Al Mutair, A. (2020). Clinical characteristics of non-intensive care unit COVID-19 patients in Saudi Arabia: A descriptive cross-sectional study. *Journal of Infection and Public Health*, *13*(11), 1639–1644. https://doi.org/10.1016/j.jiph.2020.09.003

Alshukry, A., Ali, H., Ali, Y., Al-Taweel, T., Abu-farha, M., AbuBaker, J., … Abbas, M. B. (2020). Clinical characteristics of Coronavirus Disease 2019 (COVID-19) patients in Kuwait. *MedRxiv*, 2020.06.14.20131045. https://doi.org/10.1101/2020.06.14.20131045

Alvarado, G. R., Pierson, B. C., Teemer, E. S., Gama, H. J., Cole, R. D., & Jang, S. S. (2020). Symptom Characterization and Outcomes of Sailors in Isolation After a COVID-19 Outbreak on a US Aircraft Carrier. *JAMA Network Open*, *3*(10). https://doi.org/10.1001/jamanetworkopen.2020.20981

Ayed, M., Borahmah, A., Yazdani, A., Sultan, A., Mossad, A., & Rawdhan, H. (2020). Assessment of clinical characteristics and mortality-associated factors in COVID-19 Critical cases in Kuwait. *MedRxiv*, 2020.06.17.20134007. https://doi.org/10.1101/2020.06.17.20134007

Barry, M., AlMohaya, A., AlHijji, A., Akkielah, L., AlRajhi, A., Almajid, F., … Memish, Z. A. (2020). Clinical Characteristics and Outcome of Hospitalized COVID-19 Patients in a MERS-CoV Endemic Area. *Journal of Epidemiology and Global Health*, *10*(3), 214–221. https://doi.org/10.2991/jegh.k.200806.002

Berenguer, J., Ryan, P., Rodríguez-Baño, J., Jarrín, I., & Carratalà, J. (2020). Characteristics and predictors of death among 4035 consecutively hospitalized patients with COVID-19 in Spain. *Clinical Microbiology and Infection*, *0*(0). https://doi.org/10.1016/j.cmi.2020.07.024

Bergquist, S. H., Partin, C., Roberts, D. L., O'Keefe, J. B., Tong, E. J., Zreloff, J., … Moore, M. A. (2020). Non-hospitalized Adults with COVID-19 Differ Noticeably from Hospitalized Adults in Their Demographic, Clinical, and Social Characteristics. *Sn Comprehensive Clinical Medicine*, 1–9. https://doi.org/10.1007/s42399-020-00453-3

Bernheim, A., Mei, X., Huang, M., Yang, Y., Fayad, Z. A., Zhang, N., … Chung, M. (2020). Chest CT Findings in Coronavirus Disease-19 (COVID-19): Relationship to Duration of Infection. *Radiology*, *295*(3), 200463. https://doi.org/10.1148/radiol.2020200463

Brendish, N. J., Poole, S., Naidu, V. V., Mansbridge, C. T., Norton, N., Borca, F., … Clark, T. W. (2020). Clinical characteristics, symptoms and outcomes of 1054 adults presenting to hospital with suspected COVID-19: A comparison of patients with and without SARS-CoV-2 infection. *The Journal of Infection*. https://doi.org/10.1016/j.jinf.2020.09.033

Cai, Y., Liu, J., Yang, H., Wang, M., Guo, Q., Huang, D., … Xiao, E. (2020). Association between incubation period and clinical characteristics of patients with COVID-19. *The Journal of International Medical Research*, *48*(9). https://doi.org/10.1177/0300060520956834

Cao, J., Tu, W.-J., Cheng, W., Yu, L., Liu, Y.-K., Hu, X., & Liu, Q. (2020). Clinical Features and Short-term Outcomes of 102 Patients with Corona Virus Disease 2019 in Wuhan, China. *Clinical Infectious Diseases*. https://doi.org/10.1093/cid/ciaa243

Cao, Z., Li, T., Liang, L., Wang, H., Wei, F., Meng, S., … Jin, R. (2020). Clinical characteristics of Coronavirus Disease 2019 patients in Beijing, China. *PLoS ONE*, *15*(6). https://doi.org/10.1371/journal.pone.0234764

Casas-Rojo, J. M., Antón-Santos, J. M., Millán-Núñez-Cortés, J., Lumbreras-Bermejo, C., Ramos-Rincón, J. M., Roy-Vallejo, E., … Gómez-Huelgas, R. (2020). Clinical characteristics of patients hospitalized with COVID-19 in Spain: Results from the SEMI-COVID-19 Registry. *Revista Clinica Espanola*. https://doi.org/10.1016/j.rceng.2020.07.003

Chang, M. C., Park, Y.-K., Kim, B.-O., & Park, D. (2020). Risk factors for disease progression in COVID-19 patients. *BMC Infectious Diseases*, *20*(1), 445. https://doi.org/10.1186/s12879-020-05144-x

Chen, Fang-fang, Zhong, M., Liu, Y., Zhang, Y., Zhang, K., Su, D., … Zhang, Y. (2020). The characteristics and outcomes of 681 severe cases with COVID-19 in China. *Journal of Critical Care*, *60*, 32–37. https://doi.org/10.1016/j.jcrc.2020.07.003

Chen, Fuyang, Sun, W., Sun, S., Li, Z., Wang, Z., & Yu, L. (2020). Clinical characteristics and risk factors for mortality among inpatients with COVID-19 in Wuhan, China. *Clinical and Translational Medicine*. https://doi.org/10.1002/ctm2.40

Chen, Jing, Bai, H., Liu, J., Chen, G., Liao, Q., Yang, J., … Li, K. (2020). Distinct clinical characteristics and risk factors for mortality in female COVID-19 inpatients: A sex-stratified large-scale cohort study in Wuhan, China. *Clinical Infectious Diseases*. https://doi.org/10.1093/cid/ciaa920

Chen, Jingli, Ye, J., Li, H., Xia, Z., & Yan, H. (2020, August 12). Changes in the Clinical Characteristics of 62 Patients Who Died from Coronavirus Disease 2019 [Research Article]. https://doi.org/10.1155/2020/3280908

Chen, Jun, Qi, T., Liu, L., Ling, Y., Qian, Z., Li, T., … Lu, H. (2020). Clinical progression of patients with COVID-19 in Shanghai, China. *The Journal of Infection*, *80*(5), e1–e6. https://doi.org/10.1016/j.jinf.2020.03.004

Chen, L., Deng, C., Chen, X., Zhang, X., Chen, B., Yu, H., … Sun, X. (2020). Ocular manifestations and clinical characteristics of 535 cases of COVID-19 in Wuhan, China: A cross-sectional study. *Acta Ophthalmologica*, *n/a*(n/a). https://doi.org/10.1111/aos.14472

Chen, M., Fan, Y., Wu, X., Zhang, L., Guo, T., Deng, K., … Lu, Z. (2020). *Clinical Characteristics And Risk Factors For Fatal Outcome in Patients With 2019-Coronavirus Infected Disease (COVID-19) in Wuhan, China*. SSRN Scholarly Paper, Rochester, NY. https://doi.org/10.2139/ssrn.3546069

Chen, N., Zhou, M., Dong, X., Qu, J., Gong, F., Han, Y., … Zhang, L. (2020). Epidemiological and clinical characteristics of 99 cases of 2019 novel coronavirus pneumonia in Wuhan, China: A descriptive study. *The Lancet*, *395*(10223), 507–513. https://doi.org/10.1016/S0140-6736(20)30211-7

Chen, P., Zhang, Y., Wen, Y., Guo, J., Jia, J., Ma, Y., & Xu, Y. (2020). Epidemiological and clinical characteristics of 136 cases of COVID-19 in main district of Chongqing. *Journal of the Formosan Medical Association*, *119*(7), 1180–1184. https://doi.org/10.1016/j.jfma.2020.04.019

Chen, Q., Zheng, Z., Zhang, C., Zhang, X., Wu, H., Wang, J., … Zheng, C. (2020). Clinical characteristics of 145 patients with corona virus disease 2019 (COVID-19) in Taizhou, Zhejiang, China. *Infection*, 1–9. https://doi.org/10.1007/s15010-020-01432-5

Chen, Tao, Wu, D., Chen, H., Yan, W., Yang, D., Chen, G., … Ning, Q. (2020). Clinical characteristics of 113 deceased patients with coronavirus disease 2019: Retrospective study. *BMJ*, *368*, m1091. https://doi.org/10.1136/bmj.m1091

Chen, TieLong, Dai, Z., Mo, P., Li, X., Ma, Z., Song, S., … Xiong, Y. (2020). Clinical Characteristics and Outcomes of Older Patients with Coronavirus Disease 2019 (COVID-19) in Wuhan, China: A Single-Centered, Retrospective Study. *The Journals of Gerontology*, *75*(9), 1788–1795. https://doi.org/10.1093/gerona/glaa089

Chen, Xiaoping, Jiang, Q., Ma, Z., Ling, J., Hu, W., Cao, Q., … Zhang, Y. (2020). Clinical Characteristics of Hospitalized Patients with SARS-CoV-2 and Hepatitis B Virus Co-infection. *Virologica Sinica*, 1–4. https://doi.org/10.1007/s12250-020-00276-5

Chen, Xudan, Zhu, B., Hong, W., Zeng, J., He, X., Chen, J., … Zhang, Y. (2020). Associations of clinical characteristics and treatment regimens with the duration of viral RNA shedding in patients with COVID-19. *International Journal of Infectious Diseases*, *98*, 252–260. https://doi.org/10.1016/j.ijid.2020.06.091

Chen, Yuchen, Yang, D., Cheng, B., Chen, J., Peng, A., Yang, C., … Huang, K. (2020). Clinical Characteristics and Outcomes of Patients With Diabetes and COVID-19 in Association With Glucose-Lowering Medication. *Diabetes Care*. https://doi.org/10.2337/dc20-0660

Chen, Yuhong, Zhang, K., Zhu, G., Liu, L., Yan, X., Cai, Z., … Hu, Z. (2020). Clinical characteristics and treatment of critically ill patients with COVID-19 in Hebei. *Annals of Palliative Medicine*, *9*(4), 2118–2130. https://doi.org/10.21037/apm-20-1273

Chen, Z., Hu, J., Zhang, Z., Jiang, S., Wang, T., Shi, Z., & Zhang, Z. (2020). Caution: The clinical characteristics of COVID-19 patients at admission are changing. *MedRxiv*, 2020.03.03.20030833. https://doi.org/10.1101/2020.03.03.20030833

Chu, J., Yang, N., Wei, Y., Yue, H., Zhang, F., Zhao, J., … Zhang, H. (2020). Clinical characteristics of 54 medical staff with COVID-19: A retrospective study in a single center in Wuhan, China. *Journal of Medical Virology*. https://doi.org/10.1002/jmv.25793

Colaneri, M., Sacchi, P., Zuccaro, V., Biscarini, S., Sachs, M., Roda, S., … Bruno, R. (2020). Clinical characteristics of coronavirus disease (COVID-19) early findings from a teaching hospital in Pavia, North Italy, 21 to 28 February 2020. *Eurosurveillance*, *25*(16). https://doi.org/10.2807/1560-7917.ES.2020.25.16.2000460

Covino, M., Matteis, G. D., Santoro, M., Sabia, L., Simeoni, B., Candelli, M., … Franceschi, F. (2020). Clinical characteristics and prognostic factors in COVID-19 patients aged ≥80 years. *Geriatrics & Gerontology International*, *20*(7), 704–708. https://doi.org/10.1111/ggi.13960

Dai, H., Zhang, X., Xia, J., Zhang, T., Shang, Y., Huang, R., … Li, Y. (2020). High-resolution Chest CT Features and Clinical Characteristics of Patients Infected with COVID-19 in Jiangsu, China. *International Journal of Infectious Diseases*, *95*, 106–112. https://doi.org/10.1016/j.ijid.2020.04.003

Dai, S.-P., Zhao, X., & Wu, J. (2020). Effects of Comorbidities on the Elderly Patients with COVID-19: Clinical Characteristics of Elderly Patients Infected with COVID-19 from Sichuan, China. *The Journal of Nutrition, Health & Aging*. https://doi.org/10.1007/s12603-020-1486-1

Deng, Q., Hu, B., Zhang, Y., Wang, H., Zhou, X., Hu, W., … Zhou, Q. (2020). Suspected myocardial injury in patients with COVID-19: Evidence from front-line clinical observation in Wuhan, China. *International Journal of Cardiology*, *311*, 116–121. https://doi.org/10.1016/j.ijcard.2020.03.087

Deng, Y., Liu, W., Liu, K., Fang, Y.-Y., Shang, J., Zhou, L., … Liu, H.-G. (2020). Clinical characteristics of fatal and recovered cases of coronavirus disease 2019 in Wuhan, China: A retrospective study. *Chinese Medical Journal*, *133*(11), 1261–1267. https://doi.org/10.1097/CM9.0000000000000824

D’Silva, K. M., Serling-Boyd, N., Wallwork, R., Hsu, T., Fu, X., Gravallese, E. M., … Wallace, Z. S. (2020). Clinical characteristics and outcomes of patients with coronavirus disease 2019 (COVID-19) and rheumatic disease: A comparative cohort study from a US ‘hot spot.’ *Annals of the Rheumatic Diseases*, *79*(9), 1156–1162. https://doi.org/10.1136/annrheumdis-2020-217888

Du, Y., Tu, L., Zhu, P., Mu, M., Wang, R., Yang, P., … Xu, G. (2020). Clinical Features of 85 Fatal Cases of COVID-19 from Wuhan. A Retrospective Observational Study. *American Journal of Respiratory and Critical Care Medicine*, *201*(11), 1372–1379. https://doi.org/10.1164/rccm.202003-0543OC

Duan, L., Zhang, S., Guo, M., Zhou, E., Fan, J., Wang, X., … Jin, Y. (2020). Epidemiological and clinical characteristics in patients with SARS-CoV-2 antibody negative probable COVID-19 in Wuhan. *MedRxiv*, 2020.06.18.20134619. https://doi.org/10.1101/2020.06.18.20134619

Duanmu, Y., Brown, I. P., Gibb, W. R., Singh, J., Matheson, L. W., Blomkalns, A. L., & Govindarajan, P. (2020). Characteristics of Emergency Department Patients With COVID-19 at a Single Site in Northern California: Clinical Observations and Public Health Implications. *Academic Emergency Medicine*, *27*(6), 505–509. https://doi.org/10.1111/acem.14003

Easom, N., Moss, P., Barlow, G., Samson, A., Taynton, T., Adams, K., … Lillie, P. J. (2020). Sixty-eight consecutive patients assessed for COVID-19 infection: Experience from a UK Regional infectious diseases Unit. *Influenza and Other Respiratory Viruses*, *14*(4), 374–379. https://doi.org/10.1111/irv.12739

Favà, A., Cucchiari, D., Montero, N., Toapanta, N., Centellas, F. J., Vila-Santandreu, A., … Melilli, E. (2020). Clinical characteristics and risk factors for severe COVID-19 in hospitalized kidney transplant recipients: A multicentric cohort study. *American Journal of Transplantation*, *20*(11), 3030–3041. https://doi.org/10.1111/ajt.16246

Feng, Z., Yu, Q., Yao, S., Luo, L., Zhou, W., Mao, X., … Wang, W. (2020). Early prediction of disease progression in COVID-19 pneumonia patients with chest CT and clinical characteristics. *Nature Communications*, *11*(1), 4968. https://doi.org/10.1038/s41467-020-18786-x

Fu, S., Fu, X., Song, Y., Li, M., Pan, P., Tang, T., … Ouyang, Y. (2020). Virologic and clinical characteristics for prognosis of severe COVID-19: A retrospective observational study in Wuhan, China. *MedRxiv*, 2020.04.03.20051763. https://doi.org/10.1101/2020.04.03.20051763

García-Azorín, D., Trigo, J., Talavera, B., Martínez-Pías, E., Sierra, Á., Porta-Etessam, J., … Guerrero, Á. L. (2020). Frequency and Type of Red Flags in Patients With Covid-19 and Headache: A Series of 104 Hospitalized Patients. *Headache: The Journal of Head and Face Pain*, *60*(8), 1664–1672. https://doi.org/10.1111/head.13927

Gayam, V., Chobufo, M. D., Merghani, M. A., Lamichanne, S., Garlapati, P. R., & Adler, M. K. (2020). Clinical characteristics and predictors of mortality in African-Americans with COVID-19 from an inner-city community teaching hospital in New York. *Journal of Medical Virology*. https://doi.org/10.1002/jmv.26306

Goyal, P., Choi, J. J., Pinheiro, L. C., Schenck, E. J., Chen, R., Jabri, A., … Safford, M. M. (2020). Clinical Characteristics of Covid-19 in New York City. *New England Journal of Medicine*, *382*(24), 2372–2374. https://doi.org/10.1056/NEJMc2010419

Guan, W., Liang, W., Zhao, Y., Liang, H., Chen, Z., Li, Y., … He, J. (2020). Comorbidity and its impact on 1590 patients with COVID-19 in China: A nationwide analysis. *The European Respiratory Journal*, *55*(5). https://doi.org/10.1183/13993003.00547-2020

Guan, W., Ni, Z., Hu, Y., Liang, W., Ou, C., He, J., … Zhong, N. (2020). Clinical Characteristics of Coronavirus Disease 2019 in China. *New England Journal of Medicine*, *382*(18), 1708–1720. https://doi.org/10.1056/NEJMoa2002032

Guo, T., Shen, Q., Guo, W., He, W., Li, J., Zhang, Y., … Peng, H. (2020). Clinical Characteristics of Elderly Patients with COVID-19 in Hunan Province, China: A Multicenter, Retrospective Study. *Gerontology*, *66*(5), 467–475. https://doi.org/10.1159/000508734

Han, Y., Liu, Y., Zhou, L., Chen, E., Liu, P., Pan, X., & Lu, Y. (2020). Epidemiological Assessment of Imported Coronavirus Disease 2019 (COVID-19) Cases in the Most Affected City Outside of Hubei Province, Wenzhou, China. *JAMA Network Open*, *3*(4). https://doi.org/10.1001/jamanetworkopen.2020.6785

He, S., Zhou, K., Hu, M., Liu, C., Xie, L., Sun, S., … Chen, L. (2020). Clinical characteristics of “re-positive” discharged COVID-19 pneumonia patients in Wuhan, China. *Scientific Reports*, *10*(1), 17365. https://doi.org/10.1038/s41598-020-74284-6

He, Y., Xie, M., Zhao, J., & Liu, X. (2020). Clinical Characteristics and Outcomes of Patients with Severe COVID-19 and Chronic Obstructive Pulmonary Disease (COPD). *Medical Science Monitor*, *26*, e927212. https://doi.org/10.12659/MSM.927212

Huang, C., Wang, Y., Li, X., Ren, L., Zhao, J., Hu, Y., … Cao, B. (2020). Clinical features of patients infected with 2019 novel coronavirus in Wuhan, China. *The Lancet*, *395*(10223), 497–506. https://doi.org/10.1016/S0140-6736(20)30183-5

Huang, M., Yang, Y., Shang, F., Zheng, Y., Zhao, W., Luo, L., … Wang, W. (2020). Clinical Characteristics and Predictors of Disease Progression in Severe Patients with COVID-19 Infection in Jiangsu Province, China: A Descriptive Study. *The American Journal of the Medical Sciences*, *360*(2), 120–128. https://doi.org/10.1016/j.amjms.2020.05.038

Huang, Z., Cao, J., Yao, Y., Jin, X., Luo, Z., Xue, Y., … Ge, J. (2020). The effect of RAS blockers on the clinical characteristics of COVID-19 patients with hypertension. *Annals of Translational Medicine*, *8*(7), 430. https://doi.org/10.21037/atm.2020.03.229

Imam, Z., Odish, F., Gill, I., O’Connor, D., Armstrong, J., Vanood, A., … Halalau, A. (2020). Older age and comorbidity are independent mortality predictors in a large cohort of 1305 COVID-19 patients in Michigan, United States. *Journal of Internal Medicine*, *288*(4), 469–476. https://doi.org/10.1111/joim.13119

Izquierdo, J. L., Ancochea, J., Savana COVID-19 Research Group, & Soriano, J. B. (2020). Clinical Characteristics and Prognostic Factors for Intensive Care Unit Admission of Patients With COVID-19: Retrospective Study Using Machine Learning and Natural Language Processing. *Journal of Medical Internet Research*, *22*(10), e21801. https://doi.org/10.2196/21801

Jalili, M., Payandemehr, P., Saghaei, A., Sari, H. N., Safikhani, H., & Kolivand, P. (2020). Characteristics and Mortality of Hospitalized Patients With COVID-19 in Iran: A National Retrospective Cohort Study. *Annals of Internal Medicine*. https://doi.org/10.7326/M20-2911

Javanian, M., Bayani, M., Shokri, M., Sadeghi-Haddad-Zavareh, M., Babazadeh, A., Yeganeh, B., … Ebrahimpour, S. (2020). Clinical and laboratory findings from patients with COVID-19 pneumonia in Babol North of Iran: A retrospective cohort study. *Romanian Journal of Internal Medicine*, *58*(3), 161–167. https://doi.org/10.2478/rjim-2020-0013

Ji, D., Zhang, D., Chen, Z., Xu, Z., Zhao, P., Zhang, M., … Qin, E. (2020). *Clinical Characteristics Predicting Progression of COVID-19*. https://doi.org/10.2139/ssrn.3539674

Jin, X., Lian, J.-S., Hu, J.-H., Gao, J., Zheng, L., Zhang, Y.-M., … Yang, Y. (2020). Epidemiological, clinical and virological characteristics of 74 cases of coronavirus-infected disease 2019 (COVID-19) with gastrointestinal symptoms. *Gut*, *69*(6), 1002–1009. https://doi.org/10.1136/gutjnl-2020-320926

Ke, C., Yu, C., Yue, D., Zeng, X., Hu, Z., & Yang, C. (2020). Clinical characteristics of confirmed and clinically diagnosed patients with 2019 novel coronavirus pneumonia: A single-center, retrospective, case-control study. *Medicina Clinica*, *155*(8), 327–334. https://doi.org/10.1016/j.medcli.2020.06.055

Khan, M., Khan, H., Khan, S., & Nawaz, M. (2020). Epidemiological and clinical characteristics of coronavirus disease (COVID-19) cases at a screening clinic during the early outbreak period: A single-centre study. *Journal of Medical Microbiology*, *69*(8), 1114–1123. https://doi.org/10.1099/jmm.0.001231

Khraise, W. N., Khraise, T. W., Starling Emerald, B., & Allouh, M. Z. (2020). Epidemiologic and Clinical Characteristics of COVID-19 Patients from a Quarantine Center in a Developing Community: A Retrospective Study. *International Journal of General Medicine*, *13*, 937–944. https://doi.org/10.2147/IJGM.S276742

Killerby, M. E., Link-Gelles, R., Haight, S. C., Schrodt, C. A., England, L., Gomes, D. J., … Kimball, A. (2020). Characteristics associated with hospitalization among patients with COVID-19—Metropolitan Atlanta, Georgia, March–April 2020. *Morbidity and Mortality Weekly Report*, *69*(25), 790.

Kim, G. -u, Kim, M.-J., Ra, S. H., Lee, J., Bae, S., Jung, J., & Kim, S.-H. (2020). Clinical characteristics of asymptomatic and symptomatic patients with mild COVID-19. *Clinical Microbiology and Infection*, *26*(7), 948.e1-948.e3. https://doi.org/10.1016/j.cmi.2020.04.040

Kim, M. K., Jeon, J. H., Kim, S. W., Moon, J. S., Cho, N. H., Han, E., … Lee, J. H. (2020). The Clinical Characteristics and Outcomes of Patients with Moderate-to-Severe Coronavirus Disease 2019 Infection and Diabetes in Daegu, South Korea. *Diabetes & Metabolism Journal*, *44*(4), 602–613. https://doi.org/10.4093/dmj.2020.0146

Kluytmans-van den Bergh, M. F. Q., Buiting, A. G. M., Pas, S. D., Bentvelsen, R. G., van den Bijllaardt, W., van Oudheusden, A. J. G., … Kluytmans, J. A. J. W. (2020). Prevalence and Clinical Presentation of Health Care Workers With Symptoms of Coronavirus Disease 2019 in 2 Dutch Hospitals During an Early Phase of the Pandemic. *JAMA Network Open*, *3*(5). https://doi.org/10.1001/jamanetworkopen.2020.9673

Koleilat, I., Galen, B., Choinski, K., Hatch, A. N., Jones, D. B., Billett, H., … Lipsitz, E. (2020). Clinical characteristics of acute lower extremity deep venous thrombosis diagnosed by duplex in patients hospitalized for coronavirus disease 2019. *Journal of Vascular Surgery. Venous and Lymphatic Disorders*. https://doi.org/10.1016/j.jvsv.2020.06.012

Kuang, Y., Zhang, H., Zhou, R., Lin, S., Lin, M., Wang, J., … Ji, W. (2020). *Epidemiological and Clinical Characteristics of 944 Cases of 2019 Novel Coronavirus Infection of Non-COVID-19 Exporting City, Zhejiang, China*. https://doi.org/10.2139/ssrn.3543604

Lai, C., Yu, R., Wang, M., Xian, W., Zhao, X., Tang, Q., … Wang, F. (2020). Shorter incubation period is associated with severe disease progression in patients with COVID-19. *Virulence*, *11*(1), 1443–1452. https://doi.org/10.1080/21505594.2020.1836894

Lapostolle, F., Schneider, E., Vianu, I., Dollet, G., Roche, B., Berdah, J., … Adnet, F. (2020). Clinical features of 1487 COVID-19 patients with outpatient management in the Greater Paris: The COVID-call study. *Internal and Emergency Medicine*, *15*(5), 813–817. https://doi.org/10.1007/s11739-020-02379-z

Lechien, J. R., Chiesa-Estomba, C. M., Place, S., Van Laethem, Y., Cabaraux, P., Mat, Q., … COVID-19 Task Force of YO-IFOS. (2020). Clinical and epidemiological characteristics of 1420 European patients with mild-to-moderate coronavirus disease 2019. *Journal of Internal Medicine*, *288*(3), 335–344. https://doi.org/10.1111/joim.13089

Lee, D. J., Lockwood, J., Das, P., Wang, R., Grinspun, E., & Lee, J. M. (2020). Self-reported anosmia and dysgeusia as key symptoms of coronavirus disease 2019. *Cjem*, 1–8. https://doi.org/10.1017/cem.2020.420

Lee, J. yeon, Hong, S. W., Hyun, M., Park, J. S., Lee, J. H., Suh, Y. S., … Kim, H. ah. (2020). Epidemiological and clinical characteristics of coronavirus disease 2019 in Daegu, South Korea. *International Journal of Infectious Diseases*, *98*, 462–466. https://doi.org/10.1016/j.ijid.2020.07.017

Li, G., Deng, Q., Feng, J., Li, F., Xiong, N., & He, Q. (2020, August 10). Clinical Characteristics of Diabetic Patients with COVID-19 [Research Article]. https://doi.org/10.1155/2020/1652403

Li, Jing, Ding, J., Chen, L., Hong, L., Yu, X., Ye, E., … Sun, Q. (2020). Epidemiological and clinical characteristics of three family clusters of COVID-19 transmitted by latent patients in China. *Epidemiology and Infection*, *148*. https://doi.org/10.1017/S0950268820001491

Li, Jing, Zhang, Y., Wang, F., Liu, B., Li, H., Tang, G., … Li, J. (2020). Sex differences in clinical findings among patients with coronavirus disease 2019 (COVID-19) and severe condition. *MedRxiv*, 2020.02.27.20027524. https://doi.org/10.1101/2020.02.27.20027524

Li, Jinpeng, Gao, R., Wu, G., Wu, X., Liu, Z., Wang, H., … Wu, X. (2020). Clinical characteristics of emergency surgery patients infected with coronavirus disease 2019 (COVID-19) pneumonia in Wuhan, China. *Surgery*, *168*(3), 398–403. https://doi.org/10.1016/j.surg.2020.05.007

Li, Jitian, Chen, Z., Nie, Y., Ma, Y., Guo, Q., & Dai, X. (2020). Identification of Symptoms Prognostic of COVID-19 Severity: Multivariate Data Analysis of a Case Series in Henan Province. *Journal of Medical Internet Research*, *22*(6). https://doi.org/10.2196/19636

Li, Junli, Xu, G., Yu, H., Peng, X., Luo, Y., & Cao, C. (2020). Clinical Characteristics and Outcomes of 74 Patients With Severe or Critical COVID-19. *The American Journal of the Medical Sciences*, *360*(3), 229–235. https://doi.org/10.1016/j.amjms.2020.05.040

Li, K., Wu, J., Wu, F., Guo, D., Chen, L., Fang, Z., & Li, C. (2020). The Clinical and Chest CT Features Associated With Severe and Critical COVID-19 Pneumonia. *Investigative Radiology*, *55*(6), 327–331. https://doi.org/10.1097/RLI.0000000000000672

Li, R., Tian, J., Yang, F., Lv, L., Yu, J., Sun, G., … Ding, J. (2020). Clinical characteristics of 225 patients with COVID-19 in a tertiary Hospital near Wuhan, China. *Journal of Clinical Virology*, *127*, 104363. https://doi.org/10.1016/j.jcv.2020.104363

Li, T., Lu, L., Zhang, W., Tao, Y., Wang, L., Bao, J., … Duan, J. (2020). Clinical characteristics of 312 hospitalized older patients with COVID-19 in Wuhan, China. *Archives of Gerontology and Geriatrics*, *91*, 104185. https://doi.org/10.1016/j.archger.2020.104185

Li, Xiao, Fang, X., Bian, Y., & Lu, J. (2020). Comparison of chest CT findings between COVID-19 pneumonia and other types of viral pneumonia: A two-center retrospective study. *European Radiology*, *30*(10), 5470–5478. https://doi.org/10.1007/s00330-020-06925-3

Li, Xiaochen, Xu, S., Yu, M., Wang, K., Tao, Y., Zhou, Y., … Zhao, J. (2020). Risk factors for severity and mortality in adult COVID-19 inpatients in Wuhan. *The Journal of Allergy and Clinical Immunology*, *146*(1), 110–118. https://doi.org/10.1016/j.jaci.2020.04.006

Lian, Jiang-shan, Cai, H., Hao, S., Jin, X., Zhang, X., Zheng, L., … Yang, Y. (2020). Comparison of epidemiological and clinical characteristics of COVID-19 patients with and without Wuhan exposure. *Journal of Zhejiang University. Science. B*, 1–9. https://doi.org/10.1631/jzus.B2000112

Lian, Jiangshan, Jin, X., Hao, S., Cai, H., Zhang, S., Zheng, L., … Yang, Y. (2020). Analysis of Epidemiological and Clinical Features in Older Patients With Coronavirus Disease 2019 (COVID-19) Outside Wuhan. *Clinical Infectious Diseases*, *71*(15), 740–747. https://doi.org/10.1093/cid/ciaa242

Liu, J., Ouyang, L., Guo, P., Wu, H. sheng, Fu, P., Chen, Y. liang, … Zheng, C. sheng. (2020). Epidemiological, Clinical Characteristics and Outcome of Medical Staff Infected with COVID-19 in Wuhan, China: A Retrospective Case Series Analysis. *MedRxiv*, 2020.03.09.20033118. https://doi.org/10.1101/2020.03.09.20033118

Liu, Kai, Chen, Y., Lin, R., & Han, K. (2020). Clinical features of COVID-19 in elderly patients: A comparison with young and middle-aged patients. *The Journal of Infection*, *80*(6), e14–e18. https://doi.org/10.1016/j.jinf.2020.03.005

Liu, Kui, Fang, Y.-Y., Deng, Y., Liu, W., Wang, M.-F., Ma, J.-P., … Liu, H.-G. (2020). Clinical characteristics of novel coronavirus cases in tertiary hospitals in Hubei Province. *Chinese Medical Journal*, *133*(9), 1025–1031. https://doi.org/10.1097/CM9.0000000000000744

Liu, L., Gao, J.-Y., Hu, W., Zhang, X., Guo, L., Liu, C., … Xiao, J. (2020). Clinical characteristics of 51 patients discharged from hospital with COVID-19 in Chongqing,China. *MedRxiv*, 2020.02.20.20025536. https://doi.org/10.1101/2020.02.20.20025536

Liu, S., Luo, H., Wang, Y., Cuevas, L. E., Wang, D., Ju, S., & Yang, Y. (2020). Clinical characteristics and risk factors of patients with severe COVID-19 in Jiangsu province, China: A retrospective multicentre cohort study. *BMC Infectious Diseases*, *20*(1), 584. https://doi.org/10.1186/s12879-020-05314-x

Liu, X., Lu, S., Chen, J., Xia, L., Yang, Z., Charles, S., … Lu, H. (2020). Clinical characteristics of foreign-imported COVID-19 cases in Shanghai, China. *Emerging Microbes & Infections*, *9*(1), 1230–1232. https://doi.org/10.1080/22221751.2020.1766383

Maechler, F., Gertler, M., Hermes, J., van Loon, W., Schwab, F., Piening, B., … Seybold, J. (2020). Epidemiological and clinical characteristics of SARS-CoV-2 infections at a testing site in Berlin, Germany, March and April 2020-a cross-sectional study. *Clinical Microbiology and Infection*. https://doi.org/10.1016/j.cmi.2020.08.017

Medetalibeyoglu, A., Senkal, N., Kose, M., Catma, Y., Bilge Caparali, E., Erelel, M., … Tukek, T. (2020). Older Adults Hospitalized with COVID-19: Clinical Characteristics and Early Outcomes from a Single Center in Istanbul, Turkey. *The Journal of Nutrition, Health & Aging*, 1–10. https://doi.org/10.1007/s12603-020-1477-2

Mo, P., Xing, Y., Xiao, Y., Deng, L., Zhao, Q., Wang, H., … Zhang, Y. (2020). Clinical characteristics of refractory COVID-19 pneumonia in Wuhan, China. *Clinical Infectious Diseases: An Official Publication of the Infectious Diseases Society of America*. https://doi.org/10.1093/cid/ciaa270

Mohamud, M., Mohamed, Y., Ali, A., & Ali, B. (2020). Loss of Taste and Smell are Common Clinical Characteristics of Patients with COVID-19 in Somalia: A Retrospective Double Centre Study. *Infection and Drug Resistance*, *13*, 2631–2635. https://doi.org/10.2147/IDR.S263632

Myers, L. C., Parodi, S. M., Escobar, G. J., & Liu, V. X. (2020). Characteristics of Hospitalized Adults With COVID-19 in an Integrated Health Care System in California. *JAMA*, *323*(21), 2195–2198. https://doi.org/10.1001/jama.2020.7202

Nagura-Ikeda, M., Imai, K., Kubota, K., Noguchi, S., Kitagawa, Y., Matsuoka, M., … Maeda, T. (2020). Clinical characteristics and antibody response to SARS-CoV-2 spike 1 protein using the VITROS Anti-SARS-CoV-2 antibody tests in COVID-19 patients in Japan. *MedRxiv*, 2020.08.02.20166256. https://doi.org/10.1101/2020.08.02.20166256

Nie, S., Zhao, X., Zhao, K., Zhang, Z., Zhang, Z., & Zhang, Z. (2020). Metabolic disturbances and inflammatory dysfunction predict severity of coronavirus disease 2019 (COVID-19): A retrospective study. *MedRxiv*, 2020.03.24.20042283. https://doi.org/10.1101/2020.03.24.20042283

Niu, S., Tian, S., Lou, J., Kang, X., Zhang, L., Lian, H., & Zhang, J. (2020). Clinical characteristics of older patients infected with COVID-19: A descriptive study. *Arch Gerontol Geriatr*, 104058–104058.

Nouri-Vaskeh, M., Khalili, N., Sharifi, A., Behnam, P., Soroureddin, Z., Ade, E. A., … Baradaran, B. (2020). Clinical Characteristics of Fatal Cases of COVID-19 in Tabriz, Iran: An Analysis of 111 Patients. *Advanced Journal of Emergency Medicine*. https://doi.org/10.22114/ajem.v0i0.499

Nowak, B., Szymański, P., Pańkowski, I., Szarowska, A., Życińska, K., Rogowski, W., … Wierzba, W. (2020). Clinical characteristics and short-term outcomes of patients with coronavirus disease 2019: A retrospective single-center experience of a designated hospital in Poland. *Polish Archives of Internal Medicine*, *130*(5), 407–411. https://doi.org/10.20452/pamw.15361

Peng, Y. D., Meng, K., Guan, H. Q., Leng, L., Zhu, R. R., Wang, B. Y., … Zeng, Q. T. (2020). Clinical characteristics and outcomes of 112 cardiovascular disease patients infected by 2019-nCoV. *Zhonghua Xin Xue Guan Bing Za Zhi*, *48*(6), 450–455. https://doi.org/10.3760/cma.j.cn112148-20200220-00105

Peng Yudong, Meng Kai, He Meian, Zhu Ruirui, Guan Hongquan, Ke Zihan, … Zeng Qiutang. (2020). Clinical Characteristics and Prognosis of 244 Cardiovascular Patients Suffering From Coronavirus Disease in Wuhan, China. *Journal of the American Heart Association*, *9*(19), e016796. https://doi.org/10.1161/JAHA.120.016796

Perez-Guzman, P. N., Daunt, A., Mukherjee, S., Crook, P., Forlano, R., Kont, M. D., … Nayagam, S. (2020). Clinical characteristics and predictors of outcomes of hospitalized patients with COVID-19 in a multi-ethnic London NHS Trust: A retrospective cohort study. *Clinical Infectious Diseases*. https://doi.org/10.1093/cid/ciaa1091

Popov, G. T., Baymakova, M., Vaseva, V., Kundurzhiev, T., & Mutafchiyski, V. (2020). Clinical Characteristics of Hospitalized Patients with COVID-19 in Sofia, Bulgaria. *Vector Borne and Zoonotic Diseases*. https://doi.org/10.1089/vbz.2020.2679

Price-Haywood, E. G., Burton, J., Fort, D., & Seoane, L. (2020). Hospitalization and Mortality among Black Patients and White Patients with Covid-19. *New England Journal of Medicine*, *382*(26), 2534–2543. https://doi.org/10.1056/NEJMsa2011686

Qi, D., Yan, X., Tang, X., Peng, J., Yu, Q., Feng, L., … Xiang, J. (2020). Epidemiological and clinical features of 2019-nCoV acute respiratory disease cases in Chongqing municipality, China: A retrospective, descriptive, multiple-center study. *MedRxiv*, 2020.03.01.20029397. https://doi.org/10.1101/2020.03.01.20029397

Qi, S., Guo, H., Shao, H., Lan, S., He, Y., Tiheiran, M., & Li, H. (2020). Computed Tomography Findings and Short-term follow-up with Novel Coronavirus Pneumonia. *MedRxiv*, 2020.04.02.20042614. https://doi.org/10.1101/2020.04.02.20042614

Qi, X., Liu, C., Jiang, Z., Gu, Y., Zhang, G., Shao, C., … Dong, J. (2020). Multicenter analysis of clinical characteristics and outcomes in patients with COVID-19 who develop liver injury. *Journal of Hepatology*, *73*(2), 455–458. https://doi.org/10.1016/j.jhep.2020.04.010

Qian, G.-Q., Yang, N.-B., Ding, F., Ma, A. H. Y., Wang, Z.-Y., Shen, Y.-F., … Chen, X.-M. (2020). Epidemiologic and clinical characteristics of 91 hospitalized patients with COVID-19 in Zhejiang, China: A retrospective, multi-centre case series. *QJM: An International Journal of Medicine*, *113*(7), 474–481. https://doi.org/10.1093/qjmed/hcaa089

Qin, C., Zhou, L., Hu, Z., Yang, S., Zhang, S., Chen, M., … Wang, W. (2020). Clinical Characteristics and Outcomes of COVID-19 Patients With a History of Stroke in Wuhan, China. *Stroke*, *51*(7), 2219–2223. https://doi.org/10.1161/STROKEAHA.120.030365

Qin, C., Zhou, L., Hu, Z., Zhang, S., Yang, S., Tao, Y., … Tian, D.-S. (2020). Dysregulation of immune response in patients with COVID-19 in Wuhan, China. *Clinical Infectious Diseases*. https://doi.org/10.1093/cid/ciaa248

Qin, X., Qiu, S., Yuan, Y., Zong, Y., Tuo, Z., Li, J., & Liu, J. (2020). *Clinical Characteristics and Treatment of Patients Infected with COVID-19 in Shishou, China*. https://doi.org/10.2139/ssrn.3541147

Ramos-Rincon, J.-M., Buonaiuto, V., Ricci, M., Martín-Carmona, J., Paredes-Ruíz, D., Calderón-Moreno, M., … Network, S.-C.-19. (2020). Clinical Characteristics and Risk Factors for Mortality in Very Old Patients Hospitalized With COVID-19 in Spain. *The Journals of Gerontology: Series A*. https://doi.org/10.1093/gerona/glaa243

Ruan, Q., Yang, K., Wang, W., Jiang, L., & Song, J. (2020). Clinical predictors of mortality due to COVID-19 based on an analysis of data of 150 patients from Wuhan, China. *Intensive Care Medicine*, *46*(5), 846–848. https://doi.org/10.1007/s00134-020-05991-x

Samrah, S. M., Al-Mistarehi, A.-H. W., Ibnian, A. M., Raffee, L. A., Momany, S. M., Al-Ali, M., … Khassawneh, B. Y. (2020). COVID-19 outbreak in Jordan: Epidemiological features, clinical characteristics, and laboratory findings. *Annals of Medicine and Surgery*, *57*, 103–108. https://doi.org/10.1016/j.amsu.2020.07.020

Sepulchre, E., Pittie, G., Stojkovic, V., Haesbroek, G., Crama, Y., Schyns, M., … Minon, J.-M. (2020). Covid-19: Contribution of clinical characteristics and laboratory features for early detection of patients with high risk of severe evolution. *Acta Clinica Belgica*, *0*(0), 1–7. https://doi.org/10.1080/17843286.2020.1822078

Shahriarirad, R., Khodamoradi, Z., Erfani, A., Hosseinpour, H., Ranjbar, K., Emami, Y., … Moghadami, M. (2020). Epidemiological and clinical features of 2019 novel coronavirus diseases (COVID-19) in the South of Iran. *BMC Infectious Diseases*, *20*(1), 427. https://doi.org/10.1186/s12879-020-05128-x

Shi, L., Lu, Z.-A., Que, J.-Y., Huang, X.-L., Liu, L., Ran, M.-S., … Lu, L. (2020). Prevalence of and Risk Factors Associated With Mental Health Symptoms Among the General Population in China During the Coronavirus Disease 2019 Pandemic. *JAMA Network Open*, *3*(7), e2014053–e2014053. https://doi.org/10.1001/jamanetworkopen.2020.14053

Shi, P., Ren, G., Yang, J., Li, Z., Deng, S., Li, M., … Chen, M. (2020). Clinical characteristics of imported and second-generation coronavirus disease 2019 (COVID-19) cases in Shaanxi outside Wuhan, China: A multicentre retrospective study. *Epidemiology & Infection*, *148*. https://doi.org/10.1017/S0950268820002332

Shi, Q., Zhang, X., Jiang, F., Zhang, X., Hu, N., Bimu, C., … Wang, W. (2020). Clinical Characteristics and Risk Factors for Mortality of COVID-19 Patients With Diabetes in Wuhan, China: A Two-Center, Retrospective Study. *Diabetes Care*, *43*(7), 1382–1391. https://doi.org/10.2337/dc20-0598

Shi, Q., Zhao, K., Yu, J., Jiang, F., Feng, J., Zhao, K., … Wang, W. (2020). Clinical characteristics of 101 COVID-19 nonsurvivors in Wuhan, China: A retrospective study. *MedRxiv*, 2020.03.04.20031039. https://doi.org/10.1101/2020.03.04.20031039

Shi, S. M., Bakaev, I., Chen, H., Travison, T. G., & Berry, S. D. (2020). Risk Factors, Presentation, and Course of Coronavirus Disease 2019 in a Large, Academic Long-Term Care Facility. *Journal of the American Medical Directors Association*, *21*(10), 1378-1383.e1. https://doi.org/10.1016/j.jamda.2020.08.027

Shi, S., Qin, M., Shen, B., Cai, Y., Liu, T., Yang, F., … Huang, C. (2020). Association of Cardiac Injury With Mortality in Hospitalized Patients With COVID-19 in Wuhan, China. *JAMA Cardiology*, *5*(7), 802–810. https://doi.org/10.1001/jamacardio.2020.0950

Shi, W., Gao, Z., Ding, Y., Zhu, T., Zhang, W., & Xu, Y. (2020). Clinical characteristics of COVID-19 patients combined with allergy. *Allergy*, *75*(9), 2405–2408. https://doi.org/10.1111/all.14434

Shu, L., Wang, X., Li, M., Chen, X., Ji, N., Shi, L., … Feng, G. (2020). Clinical characteristics of moderate COVID-19 patients aggravation in Wuhan Stadium Cabin Hospital: A 571 cases of retrospective cohort study. *Journal of Medical Virology*. https://doi.org/10.1002/jmv.26414

Song, C.-Y., Xu, J., He, J.-Q., & Lu, Y.-Q. (2020). COVID-19 early warning score: A multi-parameter screening tool to identify highly suspected patients. *MedRxiv*, 2020.03.05.20031906. https://doi.org/10.1101/2020.03.05.20031906

Song, F., Shi, N., Shan, F., Zhang, Z., Shen, J., Lu, H., … Shi, Y. (2020). Emerging 2019 Novel Coronavirus (2019-nCoV) Pneumonia. *Radiology*, *295*(1), 210–217. https://doi.org/10.1148/radiol.2020200274

Song, J., Hu, W., Yu, Y., Shen, X., Wang, Y., Yan, J., … Wang, M. (2020). A Comparison of Clinical Characteristics and Outcomes in Elderly and Younger Patients with COVID-19. *Medical Science Monitor*, *26*, e925047-1-e925047-8. https://doi.org/10.12659/MSM.925047

Steinmeyer, Z., Vienne-Noyes, S., Bernard, M., Steinmeyer, A., Balardy, L., Piau, A., & Sourdet, S. (2020). Acute Care of Older Patients with COVID-19: Clinical Characteristics and Outcomes. *Geriatrics*, *5*(4), 65. https://doi.org/10.3390/geriatrics5040065

Suleyman, G., Fadel, R. A., Malette, K. M., Hammond, C., Abdulla, H., Entz, A., … Brar, I. (2020). Clinical Characteristics and Morbidity Associated With Coronavirus Disease 2019 in a Series of Patients in Metropolitan Detroit. *JAMA Network Open*, *3*(6). https://doi.org/10.1001/jamanetworkopen.2020.12270

Sun, H., Ning, R., Tao, Y., Yu, C., Deng, X., Zhao, C., … Xu, D. (2020). Risk Factors for Mortality in 244 Older Adults With COVID-19 in Wuhan, China: A Retrospective Study. *Journal of the American Geriatrics Society*, *68*(6), E19–E23. https://doi.org/10.1111/jgs.16533

Sun, Ying, Dong, Y., Wang, L., Xie, H., Li, B., Chang, C., & Wang, F.-S. (2020). Characteristics and prognostic factors of disease severity in patients with COVID-19: The Beijing experience. *Journal of Autoimmunity*, *112*, 102473. https://doi.org/10.1016/j.jaut.2020.102473

Sun, Yinxiaohe, Koh, V., Marimuthu, K., Ng, O. T., Young, B., Vasoo, S., … Isais, F. S. (2020). Epidemiological and Clinical Predictors of COVID-19. *Clinical Infectious Diseases*, *71*(15), 786–792. https://doi.org/10.1093/cid/ciaa322

Tao, Y., Cheng, P., Chen, W., Wan, P., Chen, Y., Yuan, G., … Zhu, C. (2020). High incidence of asymptomatic SARS-CoV-2 infection, Chongqing, China. *MedRxiv*, 2020.03.16.20037259. https://doi.org/10.1101/2020.03.16.20037259

Tenforde, M. W., Rose, E. B., Lindsell, C. J., Shapiro, N. I., Files, D. C., Gibbs, K. W., … Gong, M. N. (2020). Characteristics of Adult Outpatients and Inpatients with COVID-19—11 Academic Medical Centers, United States, March–May 2020. *Morbidity and Mortality Weekly Report*, *69*(26), 841.

Tian, J., Yuan, X., Xiao, J., Zhong, Q., Yang, C., Liu, B., … Wang, Z. (2020). Clinical characteristics and risk factors associated with COVID-19 disease severity in patients with cancer in Wuhan, China: A multicentre, retrospective, cohort study. *The Lancet Oncology*, *21*(7), 893–903. https://doi.org/10.1016/S1470-2045(20)30309-0

Ticinesi, A., Cerundolo, N., Parise, A., Nouvenne, A., Prati, B., Guerra, A., … Meschi, T. (2020). Delirium in COVID-19: Epidemiology and clinical correlations in a large group of patients admitted to an academic hospital. *Aging Clinical and Experimental Research*, 1–8. https://doi.org/10.1007/s40520-020-01699-6

Tomlins, J., Hamilton, F., Gunning, S., Sheehy, C., Moran, E., & MacGowan, A. (2020). Clinical features of 95 sequential hospitalised patients with novel coronavirus 2019 disease (COVID-19), the first UK cohort. *The Journal of Infection*, *81*(2), e59–e61. https://doi.org/10.1016/j.jinf.2020.04.020

Vandercam, G., Simon, A., Scohy, A., Belkhir, L., Kabamba, B., Rodriguez-Villalobos, H., & Yombi, J. C. (2020). Clinical characteristics and humoral immune response in healthcare workers with COVID-19 in a teaching hospital in Belgium. *Journal of Hospital Infection*, *106*(4), 713–720. https://doi.org/10.1016/j.jhin.2020.09.018

Vena, A., Giacobbe, D. R., Di Biagio, A., Mikulska, M., Taramasso, L., De Maria, A., … Bassetti, M. (2020). Clinical characteristics, management and in-hospital mortality of patients with coronavirus disease 2019 in Genoa, Italy. *Clinical Microbiology and Infection*, *26*(11), 1537–1544. https://doi.org/10.1016/j.cmi.2020.07.049

Wan, S., Xiang, Y., Fang, W., Zheng, Y., Li, B., Hu, Y., … Yang, R. (2020). Clinical features and treatment of COVID-19 patients in northeast Chongqing. *Journal of Medical Virology*, *92*(7), 797–806. https://doi.org/10.1002/jmv.25783

Wang, A., Gao, G., Wang, S., Chen, M., Qian, F., Tang, W., … Zhang, F. (2020). Clinical Characteristics and Risk Factors of Acute Respiratory Distress Syndrome (ARDS) in COVID-19 Patients in Beijing, China: A Retrospective Study. *Medical Science Monitor*, *26*, e925974-1-e925974-9. https://doi.org/10.12659/MSM.925974

Wang, D., Hu, B., Hu, C., Zhu, F., Liu, X., Zhang, J., … Peng, Z. (2020). Clinical Characteristics of 138 Hospitalized Patients With 2019 Novel Coronavirus–Infected Pneumonia in Wuhan, China. *JAMA*, *323*(11), 1061–1069. https://doi.org/10.1001/jama.2020.1585

Wang, D., Yin, Y., Hu, C., Liu, X., Zhang, X., Zhou, S., … Peng, Z. (2020). Clinical course and outcome of 107 patients infected with the novel coronavirus, SARS-CoV-2, discharged from two hospitals in Wuhan, China. *Critical Care*, *24*(1), 188. https://doi.org/10.1186/s13054-020-02895-6

Wang, K., Kang, S., Tian, R., Zhang, X., Zhang, X., & Wang, Y. (2020). Imaging manifestations and diagnostic value of chest CT of coronavirus disease 2019 (COVID-19) in the Xiaogan area. *Clinical Radiology*, *75*(5), 341–347. https://doi.org/10.1016/j.crad.2020.03.004

Wang, L., He, W., Yu, X., Hu, D., Bao, M., Liu, H., … Jiang, H. (2020). Coronavirus disease 2019 in elderly patients: Characteristics and prognostic factors based on 4-week follow-up. *The Journal of Infection*, *80*(6), 639–645. https://doi.org/10.1016/j.jinf.2020.03.019

Wang, M., Zhang, J., Ye, D., Wang, Z., Liu, J., He, H., … Wan, J. (2020). Time-dependent changes in the clinical characteristics and prognosis of hospitalized COVID-19 patients in Wuhan, China: A retrospective study. *Clinica Chimica Acta*, *510*, 220–227. https://doi.org/10.1016/j.cca.2020.06.051

Wang, R., Pan, M., Zhang, X., Han, M., Fan, X., Zhao, F., … Shen, L. (2020). Epidemiological and clinical features of 125 Hospitalized Patients with COVID-19 in Fuyang, Anhui, China. *International Journal of Infectious Diseases*, *95*, 421–428. https://doi.org/10.1016/j.ijid.2020.03.070

Wang, S., Chen, Z., Lin, Y., Lin, L., Lin, L., Fang, S., … Shao, C. (2020). *Clinical characteristics of COVID-19 in Fujian Province: A multicenter retrospective study*. https://doi.org/10.21203/rs.3.rs-21268/v1

Wang, W., Xin, C., Xiong, Z., Yan, X., Cai, Y., Zhou, K., … Chen, J. (2020). Clinical Characteristics and Outcomes of 421 Patients With Coronavirus Disease 2019 Treated in a Mobile Cabin Hospital. *Chest*, *158*(3), 939–946. https://doi.org/10.1016/j.chest.2020.05.515

Wang, X., Fang, J., Zhu, Y., Chen, L., Ding, F., Zhou, R., … Zhao, Q. (2020). Clinical characteristics of non-critically ill patients with novel coronavirus infection (COVID-19) in a Fangcang Hospital. *Clinical Microbiology and Infection*, *26*(8), 1063–1068. https://doi.org/10.1016/j.cmi.2020.03.032

Wang, Yafei, Zhou, Y., Yang, Z., Xia, D., Hu, Y., & Geng, S. (2020). Clinical Characteristics of Patients with Severe Pneumonia Caused by the SARS-CoV-2 in Wuhan, China. *Respiration*, *99*(8), 649–657. https://doi.org/10.1159/000507940

Wang, Yang, Lu, X., Li, Y., Chen, H., Chen, T., Su, N., … Wang, J. (2020). Clinical Course and Outcomes of 344 Intensive Care Patients with COVID-19. *American Journal of Respiratory and Critical Care Medicine*, *201*(11), 1430–1434. https://doi.org/10.1164/rccm.202003-0736LE

Wang, Yi, Yao, L., Zhang, J.-P., Tang, P.-J., Ye, Z.-J., Shen, X.-H., … Yu, X. (2020). Clinical characteristics and laboratory indicator analysis of 67 COVID-19 pneumonia patients in Suzhou, China. *BMC Infectious Diseases*, *20*(1), 747. https://doi.org/10.1186/s12879-020-05468-8

Wang, Z., Yang, B., Li, Q., Wen, L., & Zhang, R. (2020). Clinical Features of 69 Cases With Coronavirus Disease 2019 in Wuhan, China. *Clinical Infectious Diseases*, *71*(15), 769–777. https://doi.org/10.1093/cid/ciaa272

Wei, C., Liu, Y., Liu, Y., Zhang, K., Su, D., Zhong, M., & Meng, X. (2020). Clinical characteristics and manifestations in older patients with COVID-19. *BMC Geriatrics*, *20*(1), 395. https://doi.org/10.1186/s12877-020-01811-5

Wen, Y., Wei, L., Li, Y., Tang, X., Feng, S., Leung, K., … Mei, S. (2020). Epidemiological and clinical characteristics of COVID-19 in Shenzhen, the largest migrant city of China. *MedRxiv*, 2020.03.22.20035246. https://doi.org/10.1101/2020.03.22.20035246

Wu, C., Chen, X., Cai, Y., Xia, J., Zhou, X., Xu, S., … Song, Y. (2020). Risk Factors Associated With Acute Respiratory Distress Syndrome and Death in Patients With Coronavirus Disease 2019 Pneumonia in Wuhan, China. *JAMA Internal Medicine*, *180*(7), 1–11. https://doi.org/10.1001/jamainternmed.2020.0994

Wu, F., Zhou, Y., Wang, Z., Xie, M., Shi, Z., Tang, Z., … Ran, P. (2020). Clinical characteristics of COVID-19 infection in chronic obstructive pulmonary disease: A multicenter, retrospective, observational study. *Journal of Thoracic Disease*, *12*(5), 1811–1823. https://doi.org/10.21037/jtd-20-1914

Wu, J., Liu, J., Zhao, X., Liu, C., Wang, W., Wang, D., … Li, L. (2020). Clinical Characteristics of Imported Cases of COVID-19 in Jiangsu Province: A Multicenter Descriptive Study. *Clinical Infectious Diseases*. https://doi.org/10.1093/cid/ciaa199

Xiao, Y., Huang, S., Yan, L., Wang, H., Wang, F., Zhou, T., … He, M. (2020). Clinical characteristics of diarrhea in 90 cases with COVID-19: A descriptive study. *Int Emerg Nurs*, 100912–100912.

Xie, J., Ding, C., Li, J., Wang, Y., Guo, H., Lu, Z., … He, H. (2020). Characteristics of patients with coronavirus disease (COVID-19) confirmed using an IgM-IgG antibody test. *Journal of Medical Virology*. https://doi.org/10.1002/jmv.25930

Xie, Y., You, Q., Wu, C., Cao, S., Qu, G., Yan, X., … Zhang, H. (2020). Impact of Cardiovascular Disease on Clinical Characteristics and Outcomes of Coronavirus Disease 2019 (COVID-19). *Circulation Journal: Official Journal of the Japanese Circulation Society*, *84*(8), 1277–1283. https://doi.org/10.1253/circj.CJ-20-0348

Xiong, S., Liu, L., Lin, F., Shi, J., Han, L., Liu, H., … Ding, S. (2020). Clinical characteristics of 116 hospitalized patients with COVID-19 in Wuhan, China: A single-centered, retrospective, observational study. *BMC Infectious Diseases*, *20*(1), 787. https://doi.org/10.1186/s12879-020-05452-2

Xu, H., Hou, K., Xu, R., Li, Z., Fu, H., Wen, L., … Zhang, N. (2020). Clinical Characteristics and Risk Factors of Cardiac Involvement in COVID-19. *Journal of the American Heart Association*, *9*(18), e016807.

Xu, T., Chen, C., Zhu, Z., Cui, M., Chen, C., Dai, H., & Xue, Y. (2020). Clinical features and dynamics of viral load in imported and non-imported patients with COVID-19. *International Journal of Infectious Diseases*, *94*, 68–71. https://doi.org/10.1016/j.ijid.2020.03.022

Xu, X.-W., Wu, X.-X., Jiang, X.-G., Xu, K.-J., Ying, L.-J., Ma, C.-L., … Li, L.-J. (2020). Clinical findings in a group of patients infected with the 2019 novel coronavirus (SARS-Cov-2) outside of Wuhan, China: Retrospective case series. *BMJ*, *368*. https://doi.org/10.1136/bmj.m606

Xu, Yang, Li, Y., Zeng, Q., Lu, Z., Li, Y., Wu, W., … Wang, X. (2020). Clinical Characteristics of SARS-CoV-2 Pneumonia Compared to Controls in Chinese Han Population. *MedRxiv*, 2020.03.08.20031658. https://doi.org/10.1101/2020.03.08.20031658

Xu, Yonghao, Xu, Z., Liu, X., Cai, L., Zheng, H., Huang, Y., … Li, Y. (2020). Clinical findings in critical ill patients infected with SARS-Cov-2 in Guangdong Province, China: A multi-center, retrospective, observational study. *MedRxiv*, 2020.03.03.20030668. https://doi.org/10.1101/2020.03.03.20030668

Yan, X., Han, X., Peng, D., Fan, Y., Fang, Z., Long, D., … Zhu, Y. (2020). Clinical Characteristics and Prognosis of 218 Patients With COVID-19: A Retrospective Study Based on Clinical Classification. *Frontiers in Medicine*, *7*. https://doi.org/10.3389/fmed.2020.00485

Yan, Y., Yang, Y., Wang, F., Ren, H., Zhang, S., Shi, X., … Dong, K. (2020). Clinical characteristics and outcomes of patients with severe covid-19 with diabetes. *BMJ Open Diabetes Research and Care*, *8*(1), e001343. https://doi.org/10.1136/bmjdrc-2020-001343

Yang, B. Y., Barnard, L. M., Emert, J. M., Drucker, C., Schwarcz, L., Counts, C. R., … Rea, T. (2020). Clinical Characteristics of Patients With Coronavirus Disease 2019 (COVID-19) Receiving Emergency Medical Services in King County, Washington. *JAMA Network Open*, *3*(7). https://doi.org/10.1001/jamanetworkopen.2020.14549

Yang, G., Tan, Z., Zhou, L., Yang, M., Peng, L., Liu, J., … He, S. (2020). Effects of Angiotensin II Receptor Blockers and ACE (Angiotensin-Converting Enzyme) Inhibitors on Virus Infection, Inflammatory Status, and Clinical Outcomes in Patients With COVID-19 and Hypertension: A Single-Center Retrospective Study. *Hypertension*, *76*(1), 51–58. https://doi.org/10.1161/HYPERTENSIONAHA.120.15143

Yang, K., Sheng, Y., Huang, C., Jin, Y., Xiong, N., Jiang, K., … Wu, G. (2020). Clinical characteristics, outcomes, and risk factors for mortality in patients with cancer and COVID-19 in Hubei, China: A multicentre, retrospective, cohort study. *The Lancet Oncology*, *21*(7), 904–913. https://doi.org/10.1016/S1470-2045(20)30310-7

Yang, Q., Xie, L., Zhang, W., Zhao, L., Wu, H., Jiang, J., … Wu, J. (2020). Analysis of the clinical characteristics, drug treatments and prognoses of 136 patients with coronavirus disease 2019. *Journal of Clinical Pharmacy and Therapeutics*, *45*(4), 609–616. https://doi.org/10.1111/jcpt.13170

Yang, W., Cao, Q., Qin, L., Wang, X., Cheng, Z., Pan, A., … Yan, F. (2020). Clinical characteristics and imaging manifestations of the 2019 novel coronavirus disease (COVID-19):A multi-center study in Wenzhou city, Zhejiang, China. *Journal of Infection*, *80*(4), 388–393. https://doi.org/10.1016/j.jinf.2020.02.016

Yang, X., Yu, Y., Xu, J., Shu, H., Xia, J., Liu, H., … Shang, Y. (2020). Clinical course and outcomes of critically ill patients with SARS-CoV-2 pneumonia in Wuhan, China: A single-centered, retrospective, observational study. *The Lancet Respiratory Medicine*, *8*(5), 475–481. https://doi.org/10.1016/S2213-2600(20)30079-5

Yao, T., Gao, Y., Cui, Q., Peng, B., Chen, Y., Li, J., … Liu, Z. (2020). Clinical characteristics of a group of deaths with COVID-19 pneumonia in Wuhan, China: A retrospective case series. *BMC Infectious Diseases*, *20*(1), 695. https://doi.org/10.1186/s12879-020-05423-7

Yao, X., Liu, S., Wang, J., Zhao, K., Long, X., He, X., … Zhang, H. (2020). The clinical characteristics and prognosis of COVID-19 patients with cerebral stroke：a retrospective study of 113 cases from one single center. *The European Journal of Neuroscience*. https://doi.org/10.1111/ejn.15007

Yu, C., Lei, Q., Li, W., Wang, X., Li, W., & Liu, W. (2020). Epidemiological and clinical characteristics of 1663 hospitalized patients infected with COVID-19 in Wuhan, China: A single-center experience. *Journal of Infection and Public Health*, *13*(9), 1202–1209. https://doi.org/10.1016/j.jiph.2020.07.002

Yu, C., Lei, Q., Li, W., Wang, X., Liu, W., Fan, X., & Li, W. (2020). Clinical Characteristics, Associated Factors, and Predicting COVID-19 Mortality Risk: A Retrospective Study in Wuhan, China. *American Journal of Preventive Medicine*, *59*(2), 168–175. https://doi.org/10.1016/j.amepre.2020.05.002

Zerah, L., Baudouin, É., Pépin, M., Mary, M., Krypciak, S., Bianco, C., … APHP / Universities / Inserm COVID-19 research collaboration. (2020). Clinical Characteristics and Outcomes of 821 Older Patients with SARS-Cov-2 Infection Admitted to Acute Care Geriatric Wards. *The Journals of Gerontology*. https://doi.org/10.1093/gerona/glaa210

Zhang, Guoxin, Nie, S., Zhang, Z., & Zhang, Z. (2020). Longitudinal Change of Severe Acute Respiratory Syndrome Coronavirus 2 Antibodies in Patients with Coronavirus Disease 2019. *The Journal of Infectious Diseases*, *222*(2), 183–188. https://doi.org/10.1093/infdis/jiaa229

Zhang, Guqin, Hu, C., Luo, L., Fang, F., Chen, Y., Li, J., … Pan, H. (2020). Clinical features and short-term outcomes of 221 patients with COVID-19 in Wuhan, China. *Journal of Clinical Virology*, *127*, 104364. https://doi.org/10.1016/j.jcv.2020.104364

Zhang, H., Shang, W., Liu, Q., Zhang, X., Zheng, M., & Yue, M. (2020). Clinical characteristics of 194 cases of COVID-19 in Huanggang and Taian, China. *Infection*, 1–8. https://doi.org/10.1007/s15010-020-01440-5

Zhang, Jie, Meng, G., Li, W., Shi, B., Dong, H., Su, Z., … Gao, P. (2020). Relationship of chest CT score with clinical characteristics of 108 patients hospitalized with COVID-19 in Wuhan, China. *Respiratory Research*, *21*(1), 180. https://doi.org/10.1186/s12931-020-01440-x

Zhang, Jishou, Wang, M., Zhao, M., Guo, S., Xu, Y., Ye, J., … Wan, J. (2020). The Clinical Characteristics and Prognosis Factors of Mild-Moderate Patients With COVID-19 in a Mobile Cabin Hospital: A Retrospective, Single-Center Study. *Frontiers in Public Health*, *8*. https://doi.org/10.3389/fpubh.2020.00264

Zhang, J.-J., Cao, Y.-Y., Tan, G., Dong, X., Wang, B.-C., Lin, J., … Gao, Y.-D. (2020). Clinical, radiological, and laboratory characteristics and risk factors for severity and mortality of 289 hospitalized COVID-19 patients. *Allergy*. https://doi.org/10.1111/all.14496

Zhang, J.-J., Dong, X., Cao, Y.-Y., Yuan, Y.-D., Yang, Y.-B., Yan, Y.-Q., … Gao, Y.-D. (2020). Clinical characteristics of 140 patients infected with SARS-CoV-2 in Wuhan, China. *Allergy*, *75*(7), 1730–1741. https://doi.org/10.1111/all.14238

Zhang, X., Cai, H., Hu, J., Lian, J., Gu, J., Zhang, S., … Yang, Y. (2020). Epidemiological, clinical characteristics of cases of SARS-CoV-2 infection with abnormal imaging findings. *International Journal of Infectious Diseases*, *94*, 81–87. https://doi.org/10.1016/j.ijid.2020.03.040

Zhang, Y., Li, H., Zhang, J., Cao, Y., Zhao, X., Yu, N., … Liu, X. (2020). The clinical characteristics and outcomes of patients with diabetes and secondary hyperglycaemia with coronavirus disease 2019: A single-centre, retrospective, observational study in Wuhan. *Diabetes, Obesity & Metabolism*, *22*(8), 1443–1454. https://doi.org/10.1111/dom.14086

Zhao, M., Wang, M., Zhang, J., Gu, J., Zhang, P., Xu, Y., … Wan, J. (2020). Comparison of clinical characteristics and outcomes of patients with coronavirus disease 2019 at different ages. *Aging (Albany NY)*, *12*(11), 10070–10086. https://doi.org/10.18632/aging.103298

Zhao, Wei, Zhong, Z., Xie, X., Yu, Q., & Liu, J. (2020). Relation Between Chest CT Findings and Clinical Conditions of Coronavirus Disease (COVID-19) Pneumonia: A Multicenter Study. *AJR. American Journal of Roentgenology*, *214*(5), 1072–1077. https://doi.org/10.2214/AJR.20.22976

Zhao, Wen, Yu, S., Zha, X., Wang, N., Pang, Q., Li, T., & Li, A. (2020). Clinical characteristics and durations of hospitalized patients with COVID-19 in Beijing: A retrospective cohort study. *MedRxiv*, 2020.03.13.20035436. https://doi.org/10.1101/2020.03.13.20035436

Zhao, X., Liu, B., Yu, Y., Wang, X., Du, Y., Gu, J., & Wu, X. (2020). The characteristics and clinical value of chest CT images of novel coronavirus pneumonia. *Clinical Radiology*, *75*(5), 335–340. https://doi.org/10.1016/j.crad.2020.03.002

Zhao, X.-Y., Xu, X.-X., Yin, H.-S., Hu, Q.-M., Xiong, T., Tang, Y.-Y., … Huang, Z.-P. (2020). Clinical characteristics of patients with 2019 coronavirus disease in a non-Wuhan area of Hubei Province, China: A retrospective study. *BMC Infectious Diseases*, *20*(1), 311. https://doi.org/10.1186/s12879-020-05010-w

Zhao, Y., Cunningham, M. H., Mediavilla, J. R., Park, S., Fitzgerald, S., Ahn, H. S., … Perlin, D. S. (2020). An Observational Study of COVID-19 from A Large Healthcare System in Northern New Jersey: Diagnosis, Clinical Characteristics, and Outcomes. *MedRxiv*, 2020.08.07.20170357. https://doi.org/10.1101/2020.08.07.20170357

Zheng, F., Tang, W., Li, H., Huang, Y.-X., Xie, Y.-L., & Zhou, Z.-G. (2020). Clinical characteristics of 161 cases of corona virus disease 2019 (COVID-19) in Changsha. *European Review for Medical and Pharmacological Sciences*, *24*(6), 3404–3410. https://doi.org/10.26355/eurrev_202003_20711

Zheng, Y., Xu, H., Yang, M., Zeng, Y., Chen, H., Liu, R., … Wang, D. (2020). Epidemiological characteristics and clinical features of 32 critical and 67 noncritical cases of COVID-19 in Chengdu. *Journal of Clinical Virology*, *127*, 104366. https://doi.org/10.1016/j.jcv.2020.104366

Zhong, Z.-F., Huang, J., Yang, X., Peng, J.-L., Zhang, X.-Y., Hu, Y., … Yang, X.-F. (2020). Epidemiological and clinical characteristics of COVID-19 patients in Hengyang, Hunan Province, China. *World Journal of Clinical Cases*, *8*(12), 2554–2565. https://doi.org/10.12998/wjcc.v8.i12.2554

Zhou, F., Yu, T., Du, R., Fan, G., Liu, Y., Liu, Z., … Cao, B. (2020). Clinical course and risk factors for mortality of adult inpatients with COVID-19 in Wuhan, China: A retrospective cohort study. *The Lancet*, *395*(10229), 1054–1062. https://doi.org/10.1016/S0140-6736(20)30566-3

Zhou, J., Sun, J., Cao, Z., Wang, W., Huang, K., Zheng, F., … Zhou, Z. (2020). Epidemiological and clinical features of 201 COVID-19 patients in Changsha city, Hunan, China. *Medicine*, *99*(34). https://doi.org/10.1097/MD.0000000000021824

Zhou, Z., Zhao, N., Shu, Y., Han, S., Chen, B., & Shu, X. (2020). Effect of Gastrointestinal Symptoms in Patients With COVID-19. *Gastroenterology*, *158*(8), 2294–2297. https://doi.org/10.1053/j.gastro.2020.03.020

Zhu, T., Wang, Y., Zhou, S., Zhang, N., & Xia, L. (2020). A Comparative Study of Chest Computed Tomography Features in Young and Older Adults With Corona Virus Disease (COVID-19). *Journal of Thoracic Imaging*, *35*(4), W97. https://doi.org/10.1097/RTI.0000000000000513
