# Supplementary material for: Clinical Features of COVID-19 Patients in the First Year of Pandemic: A Systematic Review and Meta-Analysis
Source: Biol Res Nurs. 2021 Dec 4;24(2):172–85. doi: 10.1177/10998004211055866 (PMC8968436; doi:10.1177/10998004211055866)
Supplement: sj-pdf-2-brn-10.1177_10998004211055866 – Supplemental Material for Clinical Features of COVID-19 Patients in the First Year of Pandemic: A Systematic Review and Meta-Analysis [file sj-pdf-2-brn-10.1177_10998004211055866.pdf]

Supplementary Table 2. Quality Assessment Result of Studies using the Newcastle-Ottawa Scale:

	Study	Representativeness of the sample (One Point)	Sample Size (One Point)	Non-Respondents (One Point)	Ascertainment of the exposure (One Point)	Study controls for other variable (Two	Assessment of Outcome (One Point)	Statistical Test (One Point)	Adequate Follow up time (One Point)	Score	
1	(T. Zhu et al., 2020)	0	1	0	1	1	1	1	0	5	Moderate
2	(L. Shi et al., 2020)	1	1	0	1	1	1	1	0	6	Moderate
3	(Jiangshan Lian et al., 2020)	1	1	0	1	1	1	1	0	6	Moderate
4	(Q. Yang et al., 2020)	0	1	0	1	1	1	1	0	5	Moderate
5	(X.-W. Xu et al., 2020)	0	1	0	1	1	1	1	0	5	Moderate
6	(S. Shi et al., 2020)	1	1	0	1	1	1	1	0	6	Moderate
7	(Tenforde et al., 2020)	1	1	0	1	1	1	1	0	6	Moderate
8	(Duanmu et al., 2020)	0	1	0	1	1	1	1	0	5	Moderate
9	(Bernheim et al., 2020)	1	1	1	1	1	1	1	0	7	Low
10	(Wei et al., 2020)	0	1	1	1	1	0	1	0	5	Moderate
11	(Suleyman et al., 2020)	1	1	1	1	1	1	1	0	7	Low
12	(TieLong Chen et al., 2020)	1	1	0	1	1	1	1	1	7	Low
13	(D'Silva et al., 2020)	0	1	0	1	1	1	1	0	5	Moderate
14	(Yuchen Chen et al., 2020)	1	1	0	1	1	1	1	0	6	Moderate
15	(Covino et al., 2020)	0	1	0	1	1	1	1	0	5	Moderate
16	(Q. Shi et al., 2020a)	0	1	1	1	1	0	1	0	5	Moderate
17	(H. Xu et al., 2020)	0	1	1	1	1	0	1	0	5	Moderate
18	(S. Liu et al., 2020)	1	1	0	1	1	1	1	0	6	Moderate
19	(Tao Chen et al., 2020)	0	1	1	1	1	0	1	0	5	Moderate
20	(Dawei Wang et al., 2020a)	1	1	1	1	1	1	1	0	7	Low
21	(J.-J. Zhang et al., 2020b)	1	1	0	1	1	1	1	0	6	Moderate
22	(Qingqing Chen et al., 2020)	0	1	1	1	1	0	1	0	5	Moderate
23	(F. Zheng et al., 2020)	0	1	1	1	1	0	1	0	5	Moderate
24	(H. Zhang et al., 2020)	1	1	0	1	1	1	1	0	6	Moderate
25	(Tao Li et al., 2020g)	1	1	1	1	1	1	1	0	7	Low
26	(Colaneri et al., 2020)	0	1	1	1	1	0	1	0	5	Moderate
27	(Guan et al., 2020b)	1	1	1	1	2	1	1	0	8	Low
28	(Goyal et al., 2020)	1	1	0	1	1	1	1	1	7	Low
29	(F. Wu et al., 2020)	1	1	0	1	1	1	1	0	6	Moderate
30	(Xiao et al., 2020)	0	1	1	1	1	0	1	0	5	Moderate
31	(Guo et al., 2020)	1	1	0	1	1	1	1	0	6	Moderate
32	(Y. Deng et al., 2020)	1	1	0	1	1	1	1	0	6	Moderate
33	(J. Wu et al., 2020)	0	1	1	1	1	0	1	0	5	Moderate
34	(Kui Liu et al., 2020)	0	1	0	1	1	1	1	0	5	Moderate
35	(Niu et al., 2020)	0	1	1	1	1	0	1	0	5	Moderate
36	(X.-Y. Zhao et al., 2020)	0	1	1	1	1	0	1	0	5	Moderate
37	(B. Y. Yang et al., 2020)	0	1	1	1	1	0	1	0	5	Moderate
38	(Yafei Wang et al., 2020j)	0	1	0	1	2	1	1	0	6	Moderate
39	(Mo et al., 2020)	0	1	0	1	1	1	1	0	5	Moderate
40	(Yang Xu et al., 2020)	0	1	1	1	1	1	1	0	6	Moderate
41	(Yu et al., 2020b)	1	1	0	1	1	1	1	1	7	Low

42	(Dawei Wang et al., 2020b)	0	1	1	1	1	0	1	0	5	Moderate
43	(X. Yang et al., 2020)	0	1	1	1	1	0	1	0	5	Moderate
44	(F. Zhou et al., 2020)	1	1	1	1	1	1	1	1	8	Low
45	(T. Xu et al., 2020)	0	1	1	1	1	1	1	0	6	Moderate
46	(G. Zhang et al., 2020)	0	1	0	1	2	1	1	0	6	Moderate
47	(J. Cao et al., 2020)	0	1	1	1	1	0	1	0	5	Moderate
48	(Wan et al., 2020)	0	1	0	1	2	1	1	0	6	Moderate
49	(Zhongliang Wang et al., 2020k)	0	1	1	1	1	0	1	0	5	Moderate
50	(K. Liu et al., 2020)	0	1	1	1	1	0	1	0	5	Moderate
51	(C. Huang et al., 2020)	0	1	0	1	1	1	1	0	5	Moderate
52	(Yonghao Xu et al., 2020)	0	1	1	1	1	1	1	0	6	Moderate
53	(Jun Chen et al., 2020)	1	1	0	1	1	1	1	1	7	Low
54	(Xiao Li et al., 2020h)	0	1	0	1	2	1	1	0	6	Moderate
55	(S. Qi et al., 2020)	0	1	0	1	1	1	1	0	5	Moderate
56	(Lang Wang et al., 2020e)	1	1	1	1	1	1	1	0	7	Low
57	(C.-Y. Song et al., 2020)	0	1	0	1	2	1	1	0	6	Moderate
58	(Ticinesi et al., 2020)	0	1	0	1	1	1	1	0	5	Moderate
59	(C. Qin et al., 2020b)	1	1	1	1	1	1	1	0	7	Low
60	(F. Song et al., 2020)	0	1	0	1	1	1	1	0	5	Moderate
61	(Nanshan Chen et al., 2020)	0	1	0	1	2	1	1	0	6	Moderate
62	(Peng Chen et al., 2020)	0	1	0	1	2	1	1	0	6	Moderate
63	(Shahriarirad et al., 2020)	0	1	0	1	1	1	1	0	5	Moderate
64	(D. Qi et al., 2020)	1	1	0	1	1	1	1	0	6	Moderate
65	(Han et al., 2020)	0	1	0	1	2	1	1	0	6	Moderate
66	(Jin et al., 2020)	1	1	1	1	1	1	1	0	7	Low
67	(X. Zhang et al., 2020)	0	1	1	1	2	1	1	0	7	Low
68	(García-Azorín et al., 2020)	0	1	1	1	1	1	1	0	6	Moderate
69	(Tao et al., 2020)	0	1	0	1	1	1	1	0	5	Moderate
70	(Jitian Li et al., 2020b)	0	1	0	1	1	1	1	0	5	Moderate
71	(G. Zhang et al., 2020)	0	1	0	1	2	1	1	0	6	Moderate
72	(Nie et al., 2020)	0	1	1	1	1	1	1	0	6	Moderate
73	(Imam et al., 2020)	1	1	1	1	2	1	1	0	8	Low
74	(Kluytmans-van den Bergh et al., 2020)	0	1	1	1	1	1	1	0	6	Moderate
75	(Wei Zhao et al., 2020)	0	0	1	1	2	1	1	0	6	Moderate
76	(C. Wu et al., 2020)	0	1	1	1	1	1	1	0	6	Moderate
77	(Chang et al., 2020)	0	1	0	1	1	1	1	0	5	Moderate
78	(H. Sun et al., 2020)	0	1	1	1	1	1	1	0	6	Moderate
79	(S. M. Shi et al., 2020)	0	0	1	1	2	1	1	0	6	Moderate
80	(D. J. Lee et al., 2020)	0	1	0	1	1	1	1	0	5	Moderate
81	(Easom et al., 2020)	0	1	0	1	1	1	1	0	5	Moderate
82	(Alvarado et al., 2020)	1	1	1	1	1	1	1	0	7	Low
83	(X. Zhao et al., 2020)	0	1	0	1	1	1	1	0	5	Moderate
84	(Kunhua Li et al., 2020)	0	1	1	1	1	1	1	0	6	Moderate
85	(X. Wang et al., 2020)	1	1	0	1	1	1	1	1	7	Low
86	(Javanian et al., 2020)	0	1	0	1	1	1	1	0	5	Moderate
87	(Kim et al. 2020)	0	1	1	1	1	1	1	0	6	Moderate
88	(Berenguer et al., 2020)	1	1	1	1	2	1	1	0	8	Low
89	(Maechler et al., 2020)	0	1	1	1	1	1	1	0	6	Moderate
90	(Koleilat et al., 2020)	0	1	0	1	1	1	1	0	5	Moderate
91	(K. Wang et al., 2020d)	0	1	1	1	1	0	1	0	5	Moderate
92	(Wei Wang et al., 2020i)	0	1	1	1	1	1	1	0	6	Moderate

93	(M. Huang et al., 2020)	0	1	1	1	1	0	1	0	5	Moderate
94	(J. Li et al., 2020)	0	1	0	1	1	1	1	0	5	Moderate
95	(Menglong Wang et al., 2020f)	1	1	1	1	1	1	1	0	7	Low
96	(Ke et al., 2020)	0	1	1	1	1	1	1	0	6	Moderate
97	(Jinpeng Li et al., 2020)	0	1	0	1	1	1	1	0	5	Moderate
98	(Xiaochen Li et al., 2020i)	0	1	1	1	1	1	1	0	6	Moderate
99	(Brendish et al., 2020)	0	1	0	1	1	1	1	0	5	Moderate
100	(Q. Deng et al., 2020)	0	1	1	1	1	0	1	0	5	Moderate
101	(Akbariqomi et al., 2020)	0	1	1	1	1	1	1	0	6	Moderate
102	(Vandercam et al., 2020)	0	1	0	1	1	1	1	0	5	Moderate
103	(Fang-fang Chen et al., 2020)	1	1	1	1	1	1	1	0	7	Low
104	(Ying Sun et al., 2020)	0	1	0	1	1	1	1	0	5	Moderate
105	(Ruirui Wang et al., 2020g)	0	1	1	1	1	0	1	0	5	Moderate
106	(X. Chen et al., 2020)	0	1	1	1	1	1	1	0	6	Moderate
107	(J. yeon Lee et al., 2020)	1	1	1	1	1	1	1	0	7	Low
108	(Zheng et al., 2020)	0	1	1	1	1	0	1	0	5	Moderate
109	(J. Tian et al., 2020)	1	1	1	1	2	1	1	0	8	Low
110	(K. Yang et al., 2020)	0	1	1	1	1	1	1	0	6	Moderate
111	(Akter et al., 2020)	0	1	1	1	1	1	1	0	6	Moderate
112	(Yu et al., 2020a)	1	1	1	1	1	1	1	0	7	Low
113	(Al-Omari et al., 2020)	0	1	1	1	1	0	1	0	5	Moderate
114	(Samrah et al., 2020)	0	1	0	1	1	1	1	0	5	Moderate
115	(Almazeedi et al., 2020)	1	1	1	1	1	1	1	0	7	Low
116	(Ruan et al., 2020)	0	1	1	1	1	1	1	0	6	Moderate
117	(Peng et al., 2020)	0	1	0	1	1	1	1	0	5	Moderate
118	(S Wang et al., 2020h)	0	1	1	1	1	0	1	0	5	Moderate
119	(Nouri-Vaskeh et al., 2020)	0	1	1	1	1	0	1	0	5	Moderate
120	(Khan et al., 2020)	0	1	1	1	1	1	1	0	6	Moderate
121	(L. Liu et al., 2020)	0	1	1	1	1	0	1	0	5	Moderate
122	(Jing Li et al., 2020b)	0	1	0	1	1	1	1	0	5	Moderate
123	(Zhaowei Chen et al., 2020)	0	1	1	1	1	0	1	0	5	Moderate
124	(Q. Shi et al., 2020b)	0	1	1	1	1	0	1	0	5	Moderate
125	(J. Liu et al., 2020)	0	1	1	1	1	0	1	0	5	Moderate
126	(Jingli Chen et al., 2020)	0	1	0	1	1	1	1	0	5	Moderate
127	(T. Yao et al., 2020)	0	1	1	1	1	0	1	0	5	Moderate
128	(Chen et al. 2020)	0	1	1	1	1	1	1	0	6	Moderate
129	(W. Zhao et al., 2020)	0	1	1	1	1	0	1	0	5	Moderate
130	(Wen et al., 2020)	0	1	0	1	1	1	1	0	5	Moderate
131	(Fu et al., 2020)	0	1	0	1	1	1	1	0	5	Moderate
132	(P. Shi et al., 2020)	0	1	1	1	1	0	1	0	5	Moderate
133	(Alshukry et al., 2020)	0	1	1	1	1	1	1	0	6	Moderate
134	(Duan et al., 2020)	0	1	0	1	2	1	1	0	6	Moderate
135	(Ayed et al., 2020)	0	1	1	1	1	0	1	0	5	Moderate
136	(Nagura-Ikeda et al., 2020)	0	1	0	1	2	1	1	0	6	Moderate
137	(Y. Zhao et al., 2020)	1	1	1	1	1	1	1	0	7	Low
138	(Yan et al., 2020)	0	1	1	1	1	1	1	0	6	Moderate
139	(Guozhen Li et al., 2020)	0	1	0	1	1	1	1	0	5	Moderate
140	(Guan et al., 2020)	1	1	1	1	2	1	1	0	8	Low
141	(Cai et al., 2020, p. 19)	0	1	1	1	1	0	1	0	5	Moderate
142	(Y. Xie et al., 2020)	0	1	1	1	1	0	1	0	5	Moderate
143	(M. Zhao et al., 2020)	1	1	1	1	1	1	1	0	7	Low
144	(Lai et al., 2020)	0	1	0	1	1	1	1	0	5	Moderate
145	(Favà et al., 2020)	0	1	1	1	1	1	1	0	6	Moderate
146	(J.-J. Zhang et al., 2020a)	0	1	0	1	1	1	1	0	5	Moderate

147	(W. Shi et al., 2020)	0	1	1	1	1	0	1	0	5	Moderate
148	(Z. Huang et al., 2020)	0	1	1	1	1	1	1	0	6	Moderate
149	(Bergquist et al., 2020)	0	1	0	1	1	1	1	0	5	Moderate
150	(Jalili et al., 2020)	1	1	1	1	1	1	1	0	7	Low
151	(Xiaoping Chen et al., 2020)	0	1	0	1	1	1	1	0	5	Moderate
152	(Yinxiaohe Sun et al., 2020)	0	1	0	1	1	1	1	0	5	Moderate
153	(Jing Chen et al., 2020)	1	1	1	1	1	1	1	0	7	Low
154	(Perez-Guzman et al., 2020)	0	1	1	1	1	1	1	0	6	Moderate
155	(X. Liu et al., 2020)	0	1	1	1	1	0	1	0	5	Moderate
156	(Yuhong Chen et al., 2020)	0	1	0	1	1	1	1	0	5	Moderate
157	(Sepulchre et al., 2020)	0	1	1	1	1	0	1	0	5	Moderate
158	(Fuyang Chen et al., 2020)	0	1	1	1	1	0	1	0	5	Moderate
159	(S.-P. Dai et al., 2020)	0	1	0	1	1	1	1	0	5	Moderate
160	(M. K. Kim et al., 2020)	1	1	1	1	1	1	1	0	7	Low
161	(Y. Zhang et al., 2020)	0	1	0	1	1	1	1	0	5	Moderate
162	(Y. Yan et al., 2020)	0	1	1	1	1	0	1	0	5	Moderate
163	(X. Yao et al., 2020)	1	1	1	1	1	1	1	0	7	Low
164	(Jing Li et al., 2020a)	0	1	0	1	1	1	1	0	5	Moderate
165	(J. Zhou et al., 2020)	0	1	1	1	1	1	1	0	6	Moderate
166	(J. Zhang et al., 2020)	1	1	1	1	1	1	1	0	7	Low
167	(Steinmeyer et al., 2020)	0	1	1	1	1	0	1	0	5	Moderate
168	(Zerah et al., 2020)	0	1	1	1	1	1	1	0	6	Moderate
169	(Ramos-Rincon et al., 2020)	1	1	1	1	2	1	1	0	8	Low
170	(G.-Q. Qian et al., 2020)	0	1	0	1	1	1	1	0	5	Moderate
171	(H. Dai et al., 2020)	0	1	1	1	1	0	1	0	5	Moderate
172	(G. Yang et al., 2020)	0	1	0	1	1	1	1	0	5	Moderate
173	(Mohamud et al., 2020)	0	1	1	1	1	0	1	0	5	Moderate
174	(Khraise et al., 2020)	0	1	0	1	1	1	1	0	5	Moderate
175	(Peng Yudong et al., 2020)	0	1	1	1	1	0	1	0	5	Moderate
176	(Myers et al., 2020)	0	1	1	1	1	1	1	0	6	Moderate
177	(Barry et al., 2020)	0	1	0	1	1	1	1	0	5	Moderate
178	(Almalki et al., 2020)	0	1	0	1	1	1	1	0	5	Moderate
179	(Chu et al., 2020)	0	1	0	1	1	1	1	0	5	Moderate
180	(J. Xie et al., 2020)	0	1	1	1	1	0	1	0	5	Moderate
181	(Gayam et al., 2020)	0	1	1	1	1	1	1	0	6	Moderate
182	(Shu et al., 2020)	0	1	1	1	1	1	1	0	6	Moderate
183	(Lechien et al., 2020)	1	1	1	1	1	1	1	0	7	Low
184	(Z. Cao et al., 2020)	0	1	0	1	1	1	1	0	5	Moderate
185	(Lapostolle et al., 2020)	0	1	1	1	1	1	1	0	6	Moderate
186	(Ruoqing Li et al., 2020)	0	1	0	1	1	1	1	0	5	Moderate
187	(Casas-Rojo et al., 2020)	1	1	1	1	2	1	1	0	8	Low
188	(Z. Zhou et al., 2020)	0	1	1	1	1	0	1	0	5	Moderate
189	(X. Qi et al., 2020)	0	1	0	1	1	1	1	0	5	Moderate
190	(Medetalibeyoglu et al., 2020)	0	1	1	1	1	1	1	0	6	Moderate
191	(Song et al., 2020)	0	1	1	1	1	0	1	0	5	Moderate
192	(Aibin Wang et al., 2020a)	0	1	0	1	1	1	1	0	5	Moderate
193	(Y. He et al., 2020)	0	1	1	1	1	1	1	0	6	Moderate
194	(Killerby et al., 2020)	0	1	1	1	1	1	1	0	6	Moderate
195	(Price-Haywood et al., 2020)	1	1	1	1	1	1	1	0	7	Low
196	(Nowak et al., 2020)	0	1	0	1	1	1	1	0	5	Moderate
197	(Izquierdo et al., 2020)	1	1	1	1	2	1	1	0	8	Low
198	(Vena et al., 2020)	0	1	1	1	1	0	1	0	5	Moderate
199	(W. Yang et al., 2020)	0	1	0	1	1	1	1	0	5	Moderate
200	(Tomlins et al., 2020)	0	1	1	1	1	1	1	0	6	Moderate

201	(Du et al., 2020)	0	1	1	1	1	1	1	0	6	Moderate
202	(Yang Wang et al., 2020)	0	1	0	1	1	1	1	0	5	Moderate
203	(J. Zhang et al., 2020)	0	1	1	1	1	1	1	0	6	Moderate
204	(Feng et al., 2020)	0	1	1	1	1	1	1	0	6	Moderate
205	(S. He et al., 2020)	0	1	0	1	1	1	1	0	5	Moderate
206	(Ji et al., 2020)	0	1	1	1	1	1	1	0	6	Moderate
207	(X. Qin et al., 2020)	0	1	0	1	1	1	1	0	5	Moderate
208	(Kuang et al., 2020)	0	1	0	1	1	1	1	0	5	Moderate
209	(Ming Chen et al., 2020)	0	1	1	1	1	1	1	0	6	Moderate
210	(Yi Wang et al., 2020)	0	1	0	1	1	1	1	0	5	Moderate
211	(C. Qin et al., 2020a)	1	1	1	1	1	1	1	0	7	Low
212	(Popov et al., 2020)	0	1	0	1	1	1	1	0	5	Moderate
213	(Zhong et al., 2020)	0	1	1	1	1	0	1	0	5	Moderate
214	(Xiong et al., 2020)	0	1	0	1	1	1	1	0	5	Moderate
215	(Jiang-shan Lian et al., 2020)	0	1	1	1	1	1	1	0	6	Moderate
